# Aligning With the Goals of the Planetary Health Concept Regarding Ecological Sustainability and Digital Health: Scoping Review

**DOI:** 10.2196/71795

**Published:** 2025-05-28

**Authors:** Mathea Berger, Jan Peter Ehlers, Julia Nitsche

**Affiliations:** 1 Department of Human Medicine, Division of Didactics and Educational Research in Health Care Faculty of Health Witten/Herdecke University Witten Germany

**Keywords:** climate change, digitalization, sustainability, Planetary Health, environmental protection, artificial intelligence, AI, PRISMA

## Abstract

**Background:**

Climate change, driven by greenhouse gas emissions, threatens human health and biodiversity. While the digitalization of health care, including telemedicine and artificial intelligence, offers sustainability benefits, it also raises concerns about energy use and electronic waste. Balancing these factors is key to a sustainable health care future.

**Objective:**

The objective of this review was to examine the extent to which digitalization in the health care sector influences environmental sustainability. Specifically, it aimed to assess how digitalization can contribute to reducing the health care sector’s impact on global climate change. From these findings, conclusions were drawn regarding the extent to which digitalization aligns with the objectives of the Planetary Health movement and how these 2 movements may mutually reinforce each other.

**Methods:**

A scoping review guided by the PRISMA (Preferred Reporting Items for Systematic Reviews and Meta-Analyses) 2020 guidelines using databases such as PubMed and Scopus was conducted, and 58 quantitative studies from 2009 to 2024 were analyzed for environmental, social, and economic outcomes aligned with Planetary Health goals.

**Results:**

This review analyzed 58 studies on the environmental impact of digitalization in health care primarily focusing on telemedicine, which was examined in 91% (53/58) of the studies. Most studies (56/58, 97%) quantified transport-related emissions avoided through digitalization, with some also assessing emissions from health care facilities, medical equipment, and energy consumption. Findings indicated that telemedicine significantly reduces carbon dioxide emissions, with total avoided emissions amounting to approximately 830 million kg. A substantial proportion of the studies (36/58, 62%) focused on social aspects, highlighting factors such as patient satisfaction, time efficiency, and overall convenience. In addition, economic considerations were analyzed in 48% (28/58) of the studies, emphasizing cost reductions and resource optimization. However, only 12% (7/58) of the studies evaluated the full life cycle impact of digital technologies, highlighting the need for further research on their long-term environmental sustainability.

**Conclusions:**

This review calls for further research beyond telemedicine, advocating for life cycle analyses and actionable strategies for a sustainable digitalization in health care systems. The Planetary Health framework is highlighted as a guide for ensuring sustainable digital transformation in health care.

## Introduction

### Health Care and Global Climate Change: Part of the Problem?

Human-made climate change poses a global existential threat, bringing with it several challenges and problems. Extreme weather events, loss of biodiversity, and rising sea levels are just some of the many consequences of climate change [1]. The primary cause for these catastrophic effects on the environment is the steadily rising mean near-surface temperature of the Earth. According to the Copernicus Climate Change Service, in September 2024, this was 1.54 °C above preindustrial levels [2]. The major drivers of these developments are greenhouse gas emissions, particularly carbon dioxide (CO_2_) [3]. The environmental changes associated with climate change affect human physical and mental health in various ways [4]. Air pollution and increased exposure to extreme heat affect the respiratory and cardiovascular systems [5]. Environmental changes manifest in forms such as malnutrition, increasing rates of infectious diseases [6,7], and allergies, to name a few other health consequences [8].

However, the health sector is not only confronted with consequences for human health; it also contributes significantly to climate change itself, accounting for 4.4% of global annual CO_2_ emissions [9]. These emissions are primarily caused by the direct energy requirements of health care facilities [10]. Furthermore, indirect emissions from the health care sector, including supply chains as well as the transportation of patients and staff to and from health care facilities, are particularly significant [11]. Considering options to face this problem within the health care sector, digitalization is a promising approach to not only meet the challenges of climate change but also make the health care sector as a whole more sustainable [12].

### Digitalization in Health Care: Opportunity and Additional Risk

Taking a closer look at digitalization in the health care sector, the use of telemedicine, artificial intelligence (AI), digital hospital information systems, and various other digital applications in the health care sector can be divided into 3 categories in relation to climate change according to Rahimi-Ardabili et al [13].

The first category is digitalization to mitigate the impact of the health care sector on climate change. The targeted use of AI to optimize energy consumption, for example, significantly reduces the electricity consumption of health care facilities [14]. Savings with digital technology use in the energy supply sector are estimated to be between 8% and 8.6% by 2030 [15]. Furthermore, digitalization can facilitate faster and more accurate diagnoses of various diseases such as diabetes, cardiovascular diseases, and cancer, as well as preventive approaches and patient independence in managing diseases through technological assistance, thereby reducing resource consumption in the health care sector [16]. Digital optimization of supply chains in the health care sector also promotes greater sustainability [17]. Telemedicine and web-based consultations are associated with a significant reduction of CO_2_ emissions caused by patient transportation, with savings ranging from 0.9 to 900 kg of CO_2_ per consultation [18]. They also contribute to better accessibility of health care facilities in remote areas and reductions in long-distance patient travel [19].

The second category is digitalization of the management of infectious diseases triggered by climate change. Emerging infectious diseases are a phenomenon exacerbated by climate change, environmental pollution, and population growth [20]. Establishing new technologies is a decisive measure to counter the risk of infectious diseases triggered by climate change. Epidemiological analyses, tracking and simulation models, biosensors and geoinformation systems, and apps for smartphones [21] help diagnose outbreaks of infection at an early stage; identify risk areas; and, thus, take preventive action [13].

The third category is digitalization of the management of health risks caused by climate change. The use of digital technologies such as sensor technology and AI systems constitutes environmental monitoring systems that make it possible to monitor environmental quality and make predictions about potential health risks [22]. For instance, air or water pollution [13] can be detected early, and warnings can be issued via early warning systems [23,24]. Wearable sensors enable the assessment of individual health risks related to environmental factors. Digital technologies facilitate risk mapping to identify environmental factors impacting on health and allow for early detection of environmentally associated health incidents [13].

Although digitalization is a promising strategy to make health care more environmentally sustainable [13] and address the challenges posed by climate change impacts, the ecological impact of digitalization itself should not be overlooked [25].

The first impact is increasing energy consumption due to digitalization. CO_2_ emissions from global information and communications technologies were estimated to be approximately 3.5% in 2020 [26]. Digital technologies are also associated with increased energy consumption [27,28]. The annual share of German energy demand from information and communications technology for 2030 is projected to be 7.4%, and according to some estimates, it could reach up to 9%. The energy required for the production of these technologies is more than twice the energy consumption caused by their use [15].

The second impact is increased resource requirements due to digitalization. In addition, the production of digital technologies requires large amounts of raw materials, which is problematic [14]. The extraction of necessary natural resources (eg, iron, aluminum, gold, or mercury) leads to the destruction of the environment, loss of biodiversity, and serious health effects among workers in mining areas [29].

The third impact is the effects of digitalization with regard to problematic disposal structures. The disposal of digital technologies has a negative environmental impact, with electronic waste being the fastest-growing waste stream worldwide, reaching 62 billion kg in 2022. Digital devices such as smartphones, printers, and laptops account for 47% of this total [30]. The challenge lies not only in the sheer amount of waste but also in the difficulty of recycling materials [31] and improper disposal, leading to resource loss [30] and severe environmental pollution, particularly in regions of the Global South [25]. This mishandling has highly harmful effects on the health of workers and the local population [30,32].

When examining the digitalization of health care through the lens of ecological sustainability, it is crucial to evaluate existing frameworks designed to promote environmental sustainability in the health care sector. The central question is how effectively these frameworks can be adopted for digitalization and whether they synergize to enhance sustainability or create obstacles to each other’s implementation.

### Digital Health and Planetary Health: Complement or Conflict?

One of these efforts to make health care more sustainable is that of *Planetary Health*, which addresses the interdependencies between human health and the health of the planet. More precisely, it describes an integrative, interdisciplinary concept that considers Planetary Health at various levels—social, ecological, and economic [33]. Planetary Health addresses not only the numerous environmental changes such as climate change, deforestation, loss of biodiversity, and environmental pollution but also the health effects of these changes [34]. However, it is not just a concept; it serves as a guide for institutions to create policies that make the health care sector more environmentally sustainable [35]. Planetary Health provides an excellent basis for addressing the interactions between the environmental impacts of the health care sector and human health [36].

If we consider the ecological dilemma of digitalization described previously against the backdrop of the *Planetary Health* concept, it must be questioned to what extent digitalization efforts themselves have a negative impact on the environment and human health and how these effects can be mitigated. Initial steps such as implementations of life cycle analyses (LCAs) [37], approaches to the digital circular economy [25,31], green IT, and the development of policies and frameworks [12,38] for digitalization in terms of ecological sustainability already exist in the literature and are being discussed.

To more precisely examine how digitalization in health care relates to the sustainability goals of Planetary Health and to what extent it supports or challenges these objectives, a scoping review was conducted. In addition, this review aimed to identify knowledge gaps in the literature regarding the measurement and assessment of ecological sustainability in this field. Through a comprehensive analysis of the existing body of research, this review sought to provide insights into areas in which further investigation is necessary to better understand the environmental impacts of health care digitalization and how it aligns with the sustainability objectives outlined by the Planetary Health concept. It should be examined whether the goals of the Planetary Health concept can be applied to the digitalization of health care and whether their implementation within a framework is feasible or whether they conflict with one another.

## Methods

### Search Strategy and Selection Criteria

This scoping review followed the PRISMA (Preferred Reporting Items for Systematic Reviews and Meta-Analyses) 2020 checklist [39,40] (Multimedia Appendix 1) without being registered under a protocol. This review investigated the impact of digitalization in health care on achieving environmental sustainability goals within the Planetary Health framework. The key research questions (RQs) were as follows:

- RQ1: What are the environmental benefits of health care digitalization?
- RQ2: What potential negative environmental impacts are associated with digitalization in health care?
- RQ3: How does digitalization align with Planetary Health’s sustainability goals?

The PubMed, Scopus, and MEDLINE databases were used to identify relevant articles. Additional studies were incorporated through citation searches of pertinent literature. The search was conducted on August 14, 2024, and included all quantitative studies published up to that date. The search string comprised 3 main categories: “digitalization,” “sustainability/environmental effects,” and “healthcare,” tailored for each database using Boolean operators (Multimedia Appendix 2). The search was further restricted to titles and abstracts. An analysis of study quality was not conducted because it was prioritized to identify gaps and trends in the literature.

The search aimed to identify quantitative primary data on the environmental impact of digitalization in the health care sector. The inclusion criteria were based on the population, intervention, comparison, and outcome framework [41], requiring that studies meet all the categories shown in Textbox 1. To minimize bias from a single reviewer, the inclusion criteria were predefined in tandem.

**Inclusion criteria showing the population, intervention, comparison, and outcome categories; further descriptions; and examples.**

- Population: all relevant groups in the health care sector affected by digitalization (eg, patients, employees, and health insurers)
- Intervention: any digital technology or digital process (eg, telemedicine, electronic health records, artificial intelligence, and digital applications)
- Comparison: comparisons involving older technology or traditional versus digital approaches (eg, digital vs nondigital and environmental impact before and after digitalization)
- Outcome: environmental impact attributed to digitalization in health care (eg, carbon dioxide emissions, greenhouse gas emissions, electronic waste, waste reduction, and reduction of environmental resources)

Search results were excluded if they were books, conference proceedings, editorials, or commentaries. Reviews and overview articles were not considered during the initial screening, but their citations were screened in a second step. Studies not relevant to the topic or lacking quantitative data were also excluded. Furthermore, papers in languages other than German or English were excluded.

### Data Analysis

The studies retrieved from the literature search were uploaded to Rayyan (Qatar Computing Research Institute [42]), and duplicates were automatically removed. The studies were initially screened by a single reviewer based on titles and abstracts according to the inclusion criteria. Studies in which uncertainties regarding the fulfillment of inclusion criteria arose were discussed with a second reviewer to mitigate the risk of single-reviewer bias. Uncertainty regarding the inclusion criteria was defined as a lack of clarity in at least one population, intervention, comparison, and outcome criterion.

In cases of disagreement regarding study eligibility, the studies were initially included in the full-text screening to allow for a more thorough assessment. If consensus was reached on inclusion or exclusion, the studies were either included or excluded accordingly. Studies deemed suitable for full-text screening were uploaded to Rayyan again. The full-text screening was conducted following the same procedure as for the abstract screening. Studies for which there were disagreements regarding the fulfillment of inclusion criteria were discussed until consensus was reached. All included and excluded studies were reviewed and validated by a second reviewer to minimize the risk of single-reviewer bias.

Data were analyzed according to predefined categories developed by the 2 reviewers. The following information was compiled in a Microsoft Excel spreadsheet (Microsoft Corp): author, year, country, study design, aims, and objectives. In addition, secondary outcomes such as social and economic impacts were recorded. Social outcomes included effects on patients and staff, such as satisfaction, experience, and health outcomes. The economic impact was defined as the financial effect on the health care system or patients. These aspects were considered to analyze, in line with the holistic approach of the Planetary Health concept, how many studies already adopt a more comprehensive, integrated perspective on the topic of sustainability. Other categories included the digital technology examined, application of LCA, and the method of calculating the ecological impact of the digital technology assessed. Calculations for *nondigital* or traditional alternatives were also recorded. Finally, data on the environmental impacts were examined, and their results were compiled. The extraction was conducted by 1 reviewer and discussed collaboratively to validate the extracted data.

## Results

### Study Characteristics and Methodological Approaches

**Figure 1:**
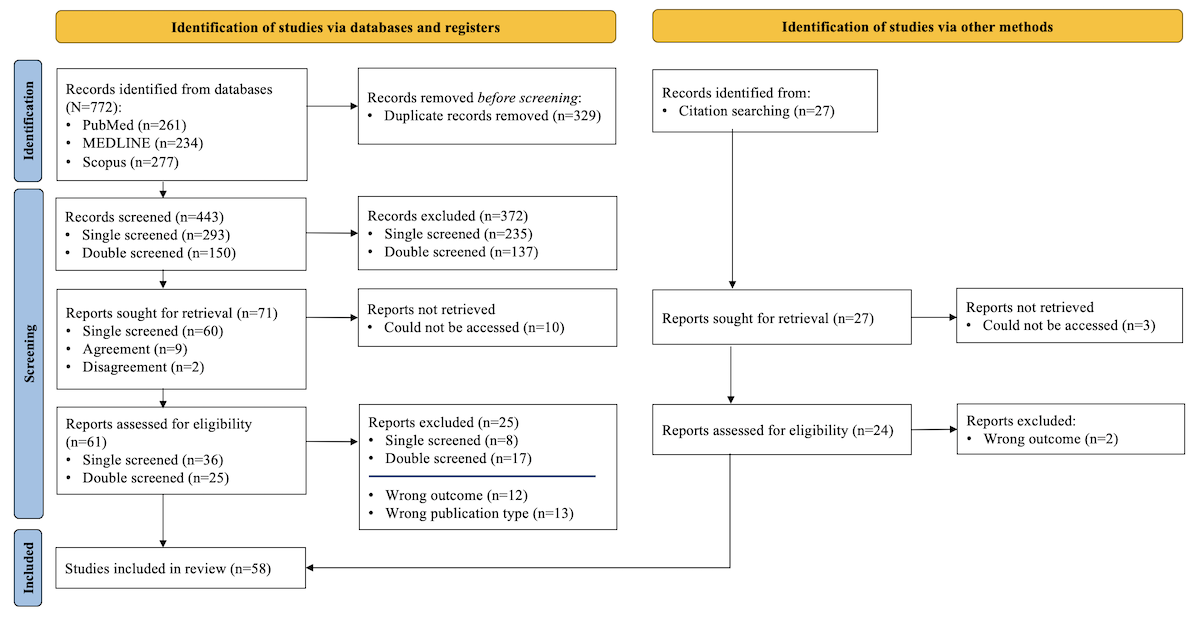
PRISMA 2020 flow diagram for new systematic reviews that includes searches of databases, registers, and other sources.

The search yielded a total of 772 hits. After excluding duplicates, of the 772 hits, 444 (57.5%) abstracts were screened, and 151 (19.6%) abstracts were double screened with the second reviewer because of uncertainties. In this step, of the 444 abstracts screened, a total of 137 (30.9%) studies were excluded jointly, and 5 (1.1%) studies were included. Due to disagreements, 2% (9/444) of the studies were included in the full-text screening, resulting in a total of 71 studies included in the full-text screening. Of these 71 studies, 10 (14%) were inaccessible and, therefore, excluded. From the database search, 59% (36/61) of the studies were included in the data extraction, and 41% (25/61) were excluded with specified reasons according to the exclusion criteria. After a collaborative discussion on 25 studies, 17 (68%) were excluded based on predefined criteria, and 8 (32%) were selected for inclusion. An additional 22 studies were identified through citation tracking. In total, 58 studies were analyzed and included in the review (Figure 1). The results, sorted by the defined categories, are provided in Table 1.

**Table 1:** Included studies analyzed by the defined categories.

Study, country, year	Setting (dept)	Aims and objectives	Digital Technology	Data^a^	Tool	Factors^b^	Tool—LCA^c^	Outcome^d^	Key findings
Curtis et al [43], United Kingdom, 2021	Hospital (orthopedics)	Evaluating the impact of NF2F^e^ versus F2F^f^ emergency orthopedic clinic referrals	Telemedicine (T^g^)	Travel-related emissions and building emissions	Travel-related emissions: UK DEFRA^h^; building emissions: NHS^i^ Sustainable Development Unit	None	NA^j^	Carbon emissions (CO_2_^k^)	Avoided emissions per NF2F consultation: 56 kg of CO_2_; avoided emissions in total for 104 NF2F consultations: 5846 kg of CO_2_ equivalent
Heffernan et al [44], Canada, 2023	Hospital (pediatrics)	Evaluating potential carbon savings of NF2F versus F2F in OSA^l^ and OM^m^with effusion surgery care	Telemedicine (VC^n^)	Travel-related emissions	Canada’s National GHG^o^ Inventory Report	EEC^p^ and OEC^q^ (D^r^)	EEC: NA; OEC: Government of British Columbia	Carbon emissions (CO_2_)	Avoided emissions per NF2F consultation: 63.86 kg of CO_2_
Holmner et al [19], Sweden, 2014	University hospital (surgery)	Evaluating the carbon footprint of NF2F hand and plastic surgery rehabilitation appointments	Telemedicine (VC)	Travel-related emissions	Lenzen [45]	EEC and OEC (D, DT^s^, and SR^t^)	EEC: LCA (Ong et al [46]); OEC: NA	Carbon emissions (CO_2_)	Avoided emissions in total for 238 NF2F consultations: 602 kg of CO_2_; 1.86-8.43 kg of CO_2_ per hour of web-based consultation
Smith et al [47], United Kingdom, 2013	Nationwide health care sector	Evaluating the carbon emissions associated with different smoking cessation support methods, including SMS text message support and telephone, group, and individual counseling	Telemedicine (NS^u^)	Travel-related emissions and building emissions	NIHR^v^	Travel-related emissions: EEC; building emissions: OEC	Travel-related emissions: Mobile’s Green Manifesto; building emissions: UK DEFRA	Carbon emissions (CO_2_)	Emissions per 1000 patients for SMS text messages: 8143 kg of CO_2_; emissions per 1000 patients for telephone counseling: 8619 kg of CO_2_; emissions per 1000 patients for group counseling: 16,114 kg of CO_2_; emissions per 1000 patients for individual counseling: 16,372 kg of CO_2_
Connor et al [48], United Kingdom, 2011	University hospital (nephrology)	Evaluating travel-related reduction in GHG emissions of telephone consultations for routine follow-up care for patients undergoing renal transplantation	Telemedicine (T)	Travel-related emissions	Google Maps and UK DEFRA	None	NA	Carbon emissions (CO_2_)	Avoided emissions in total for 350 NF2F consultations: 2818 kg of CO_2_; avoided emissions in total for 350 NF2F consultations+staff travel: 3049 kg of CO_2_
Kassa et al [49], Sweden, 2024	Hospital (pediatrics)	Evaluating NF2F versus F2F consultations according to potential travel-related carbon savings	Telemedicine (VC)	Travel-related emissions	Google Maps and online CO_2_ calculator	None	NA	Carbon emissions (CO_2_)	Avoided emissions per family: 126.3 kg of CO_2_; median 267 km traveled
Savoldelli et al [50], Italy, 2024	University hospital (cardiology)	Evaluating the environmental impact of NF2F versus F2F follow-up care	Telemedicine (VC)	Travel-related emissions, waste, and digital device	Travel-related emissions: ISO^w^ 14040/14044 and Ecological Footprint Calculator; waste: ISO 14040/14044; digital device: ecoinvent version 3.8 and Ecological Footprint Calculator	Travel-related emissions: EEC (D and SR); waste and digital device: OEC (D and SR)	Travel-related emissions: ecoinvent version 3.8 and Ecological Footprint Calculator; waste and digital device: ISO 14040/14044 and Sillcox et al [51]	Carbon emissions (CO_2_)	NF2F emissions per patient: 0.41 kg of CO_2_; F2F emissions per patient: 9.77 kg of CO_2_
Tselapedi-Sekeitto et al [52], Canada, 2024	Hospital (otolaryngology)	Evaluating the environmental impact of NF2F versus F2F consultations	Telemedicine (VC)	Travel-related emissions	US EPA^x^	None	NA	Carbon emissions (CO_2_)	Emissions per F2F consultation: mean 32 (SD 39) kg of CO_2_ equivalent; avoided emissions in total for 33 NF2F consultations: mean 1056 (SD 224) kg of CO_2_
Welk et al [53], Canada, 2022	Regional health care sector	Evaluating the environmental and patient-level financial benefits of widespread NF2F care	Telemedicine (VC)	Travel-related emissions	IEA^y^	None	NA	Carbon emissions (CO_2_)	Avoided emissions in total for 63,556,271 NF2F consultations in 22 mo (on 3,197,099,600 km traveled): 658 million kg of CO_2_; avoided emissions for 10% use of public transit: 589 million kg of CO_2_; avoided emissions for 20% use of public transit: 545 million kg of CO_2_; avoided emissions per NF2F visit for 10% use of public transit: 9.3 kg of CO_2_; avoided emissions per NF2F visit for 20% use of public transit: 8.4 kg of CO_2_
Dorrian et al [54], Scotland, 2009	Primary health care	Evaluating the feasibility and impact of NF2F versus F2F endoscopy	Telemedicine (NS)	Travel-related emissions	UK DEFRA and AA Route Planner	None	NA	Carbon emissions (CO_2_)	Avoided emissions per NF2F patient: 123 kg of CO_2_ equivalent (round trip)
Thiel et al [55], United States, 2023	University hospital (all)	Evaluating the environmental impact of NF2F versus F2F consultations	Telemedicine (VC)	Travel-related emissions, building emissions, and medical equipment	US EPA	Travel-related emissions: EEC; building emissions and medical equipment: OEC	Travel-related emissions: ISO 14040/14044, LCA SimaPro 9.3.0.240 LCI^z^ database, and ecoinvent version 3.82; building emissions and medical equipment: US EPA	Carbon emissions (CO_2_)	Emissions per NF2F consultation: 0.02-0.04 kg of CO_2_ equivalent; emissions per F2F consultation: 20 kg of CO_2_ equivalent; avoided emissions in total per y: 17,000 metric tons of CO_2_ equivalent
Morcillo Serra et al [56], Spain, 2022	Insurance company	Evaluating the environmental impact of NF2F versus F2F consultations	Telemedicine (VC) and EHR^aa^	Travel-related emissions and paper use (energy for printing)	Carbon Trust	OEC (D and DT)	Carbon Trust	GHG emissions	Avoided emissions per NF2F consultation: 3.057 kg of CO_2_ equivalent; avoided emissions in total for NF2F and download of medical reports: 6,655,000 kg of CO_2_
Jiang et al [57], United States, 2021	Nationwide health care sector (oncology)	Evaluating NF2F services and estimating the financial and environmental impacts of these services compared to F2F visits	Telemedicine (T and VC)	Travel-related emissions	Google Maps and US EPA	None	NA	Carbon emissions (CO_2_)	Avoided emissions in total for 560 NF2F consultations: 35,500 kg of CO_2_ per 3 mo (on 139,160 km traveled)
Cummins et al [58], United States, 2024	Nationwide health care sector	Evaluating the environmental impact of NF2F versus F2F consultations	Telemedicine (VC)	Travel-related emissions	US EPA and ArcGIS	OEC (D and DT)	US EPA	Carbon emissions (CO_2_)	Avoided emissions per NF2F consultation: 19.821 kg of CO_2_ equivalent; avoided emissions in total for 6,231,614 NF2F consultations: 123,461,000 kg of CO_2_
Grabski et al [59], United States, 2024	Hospital (pediatrics)	Evaluating the environmental impact of NF2F versus F2F consultations	Telemedicine (VC)	Travel-related emissions	US EPA	None	NA	GHG emissions	Avoided emissions in total for 20,845 NF2F consultations: 618,000 kg of CO_2_ (1,562,716 miles traveled), 5400 kg of carbon monoxide, 670 kg of nitric oxide, and 6100 kg of volatile organic compounds
Lewis et al [60], Wales, 2009	Hospital	Evaluating the environmental impact of NF2F versus F2F consultations	Telemedicine (VC)	Travel-related emissions	UK DEFRA	None	NA	Carbon emissions (CO_2_)	Avoided emissions in total for 21 NF2F consultations per y: 1696 kg of CO_2_; avoided emissions in total for 30 NF2F consultations per y: 2590 kg of CO_2_
Evers et al [61], United States, 2021	Hospital (infectiology)	Evaluating NF2F versus F2F impact on various costs and benefits related to health care visits	Telemedicine (VC)	Travel-related emissions	Google Maps and US EPA	None	NA	Carbon emissions (CO_2_)	Avoided emissions per NF2F patient: average of 91.79 kg of CO_2_ equivalent
Arndt et al [62], Germany, 2023	University hospital (orthopedics)	Evaluating the environmental impact of NF2F versus F2F consultations	Telemedicine (VC)	Travel-related emissions	TREMOD^ab^	OEC (SR)	UBA^ac^	Carbon emissions (CO_2_)	Emissions per NF2F consultation: 0.004 kg of CO_2_ equivalent; emissions per F2F consultation: 500 kg of CO_2_ equivalent (average of 33 km traveled); avoided emissions in total per y: 17,000 tons of CO_2_ equivalent
Cockrell et al [63], United States, 2022	Hospital (pediatrics)	Evaluating the environmental impact of NF2F versus F2F consultations	Telemedicine (VC)	Travel-related emissions	R Core Team and US EPA	None	NA	Carbon emissions (CO_2_)	Avoided emissions in total for 10,626 NF2F consultations per y: 344,000 kg of CO_2_ (1,427,497 km traveled)
Sellars et al [64], Scotland, 2020	Hospital (gastroenterology)	Evaluating the environmental impact of NF2F versus F2F consultations	Telemedicine (VC)	Travel-related emissions	AA Route Planner, UN^ad^ ICAO^ae^, and Map My Emissions	None	NA	Carbon emissions (CO_2_)	Avoided emissions per NF2F consultation: 42.27 kg of CO_2_ equivalent; avoided emissions in total for 6685 NF2F consultations: 2113.29 kg of CO_2_ equivalent
Al Fannah et al [65], Oman, 2022	Nationwide health care sector	Evaluating the environmental impact of NF2F versus F2F consultations and inhalation anesthetic gases	Telemedicine (VC)	Travel-related emissions	Ravindrane and Patel [66]	None	NA	Carbon emissions (CO_2_)	Avoided emissions per NF2F consultation: 0.69-190 kg of CO_2_ equivalent; avoided emissions in total for 18,332 NF2F consultations: 12.65-3483.1 tons of CO_2_; avoided emissions in total for 34,792 NF2F consultations: 24-6610.5 tons of CO_2_
Robinson et al [67], United States, 2017	Specialized health center (pediatrics)	Evaluating the environmental impact of NF2F versus F2F preoperative screenings for SPML^af^ surgery	Telemedicine (T)	Travel-related emissions	ArcGIS and US EPA	None	NA	GHG emissions	Avoided emissions in total for 161 NF2F patients: 43,595 kg of CO_2_ equivalent (170,703 km traveled); avoided emissions in total for 161 NF2F consultations per y: 14,532 kg of CO_2_ equivalent
Chang et al [68], United States, 2023	Hospital (surgery)	The study aimed to assess the patient cost burden and environmental impact of travel versus telehealth for general surgery outpatient visits before and during the COVID-19 pandemic	Telemedicine (VC)	Travel-related emissions	US EPA	None	NA	Carbon emissions (CO_2_)	Avoided emissions for 307 NF2F visits before the COVID-19 pandemic: 81,590 kg of CO_2_; avoided emissions for 3256 NF2F visits during the COVID-19 pandemic: 481,341 kg of CO_2_; avoided emissions for 2949 NF2F visits (difference between the 2 periods): 399,751 kg of CO_2_
Muschol et al [69], Germany, 2022	University hospital (orthopedics)	Evaluating the environmental impact of NF2F versus F2F follow-ups	Telemedicine (VC)	Travel-related emissions	UBA	None	NA	GHG emissions	Avoided emissions per NF2F consultation: GHGs—11.25 kg of CO_2_ equivalent, 0.07 kg of carbon monoxide, 0.011 kg of volatile hydrocarbons, 0.028 kg of nitrogen oxides, and 0.0004 kg of particulates; avoided emissions in total for 26 NF2F consultations: GHGs—292.45 kg of CO_2_ equivalent, 1.82 kg of carbon monoxide, 0.29 kg of volatile hydrocarbons, 0.73 kg of nitrogen oxides, and 0.01 kg of particulates
Vidal-Alaball et al [70], Spain, 2019	Nationwide health care sector	Evaluating the environmental impact of NF2F versus F2F consultations	Telemedicine (VC)	Travel-related emissions	Google Maps and Ministry of Territory, Housing, and Ecological Transition	None	NA	GHG emissions	Avoided emissions in total for 9034 NF2F consultations: 29,384 kg of CO_2_ (on 192,682 km traveled)
King et al [71], United Kingdom, 2022	Hospital (gastroenterology)	Evaluating the environmental impact of NF2F versus F2F consultations	Telemedicine (T)	Travel-related emissions	Google Maps and UK BEIS^ag^	OEC	NA	Carbon emissions (CO_2_)	Emissions per F2F consultation: 1.54 kg of CO_2_ equivalent; emissions per NF2F consultation: 0.005 kg of CO_2_ equivalent
Lee et al [72], United States, 2021	University hospital (dermatology)	Evaluating the environmental impact of NF2F versus F2F isotretinoin management visits	Telemedicine (VC)	Travel-related emissions	Google Maps, US EPA, and FHWA^ah^	None	NA	GHG emissions	Avoided e emissions for 710 NF2F consultations per y: 47,900 kg of CO_2_, 50.0 kg of methane, 41,395 kg of nitrous oxide, and 1040 kg of hydrofluorocarbons; total: 49,400 kg
Patel et al [73], United States, 2023	Specialized health center (oncology)	Evaluating the environmental impact of NF2F versus F2F consultations	Telemedicine (VC)	Travel-related emissions	US EPA	None	NA	Carbon emissions (CO_2_)	Avoided emissions per NF2F consultation with a driving time of ≤60 min: 19.8 kg of CO_2_ equivalent (on a median of 77.41 km traveled); avoided emissions per NF2F consultation with a driving time of >60 min: 98.6 kg of CO_2_ equivalent (on a median of 385.92 km traveled)
Dieperink et al [74], Denmark, 2023	University hospital (oncology)	Evaluating the environmental impact of NF2F versus F2F consultations	Telemedicine (VC)	Travel-related emissions	US EPA	None	NA	Carbon emissions (CO_2_)	Avoided emissions per NF2F consultation: 37.9 kg of CO_2_ equivalent (on a median of 120 km traveled); avoided emissions in total for 84 NF2F consultations: 3183.31 kg of CO_2_ equivalent (on 12,877 km traveled)
Schmitz-Grosz et al [75], Germany and Switzerland, 2023	Insurance company	Evaluating the environmental impact of NF2F versus F2F health care model in Switzerland	Telemedicine (VC)	Travel-related emissions	Mobitool and Federal Office for Spatial Development	OEC (D, duration, and SR) and building emissions	UBA	Carbon emissions (CO_2_)	Avoided emissions per NF2F consultation: 0.57 kg of CO_2_ equivalent (on an average of 4.15 km traveled); avoided emissions in total for 433,890 consultations: 248.48 tons of CO_2_ equivalent (on 1,800,391 km traveled)
Iaccarino et al [76], United States, 2022	University hospital (rehabilitation)	Evaluating the environmental impact and benefits of NF2F versus F2F rehabilitation visits	Telemedicine (VC)	Travel-related emissions	US EPA	None	NA	GHG emissions	Avoided emissions in total for 212 NF2F consultations: 16.67 kg of volatile organic gases, 12.94 kg of nitrogen oxides, 161.24 kg of carbon monoxide, 6993.048 kg of CO_2_; PM10^ai^: 0.067 kg; PM2.5^aj^: 0.061 kg
Barakat-Johnson et al [77], Australia, 2022	Specialized health center (dermatology)	Assessing the viability and acceptability of an eWCC^ak^ supported by a digital wound management application	Digital application	Travel-related emissions	Google maps and Green Vehicle Guide	None	NA	Carbon emissions (CO_2_)	Avoided emissions for 51 NF2F visits: 250 kg of CO_2_ (on an average of 638 km traveled)
Connor et al [78], United Kingdom, 2019	Hospital (urology)	Evaluating the environmental impact of NF2F versus F2F consultations	Telemedicine (NS)	Travel-related emissions	Google Maps and Carbon Footprint	None	NA	Carbon emissions (CO_2_)	Avoided emissions in total for 1008 NF2F consultations: 700-2930 kg of CO_2_ equivalent (on 15,085 km traveled)
Milne-Ives et al [79], United Kingdom, 2022	Hospital	Evaluating the clinical and environmental impact of MyPreOp, a cloud-based preoperative assessment platform, in comparison to traditional preoperative procedures	Web platform	Travel-related emissions	Carbon Footprint	None	NA	Carbon emissions (CO_2_)	Avoided emissions in total for 813 NF2F consultations per 4 mo: 9050 kg of CO_2_ equivalent
Moncho-Santonja et al [80], Spain, 2023	Hospital	Evaluating the environmental impact of NF2F versus F2F follow-up consultations	Telemedicine (VC)	Travel-related emissions	Google Maps	None	NA	GHG emissions	Avoided emissions per NF2F consultations (on an average of 20.7 km traveled): 3.678 kg of CO_2_ equivalent, 3.652 kg of CO_2_, 0.0034 kg of methane, and 0.0226 kg of nitrous oxide; avoided emissions in total for 21,548 NF2F consultations: 79,260.27 kg of CO_2_ equivalent, 78,699.41 kg of CO_2_, 74.52 kg of methane, and 487.27 kg of nitrous oxide
Turley et al [81], United States, 2011	Nationwide health care sector	Evaluate the environmental impact of Kaiser Permanente’s EHR system	EHR	Travel-related emissions	ArcGIS	EEC and OEC (D)	EcoHealth Footprint framework	GHGs, waste, toxic chemicals, water use, air pollutants, and use of land for buildings	Avoided emissions through paper elimination: 21,800 tons of CO_2_ per y; avoided emissions through NF2F consultations (26% reduction in in-person visits): 26,000-92,000 tons of CO_2_ per y; emissions produced by hardware and data center: 37,229,505 kg of CO_2_ equivalent; avoided plastic waste by digitalizing x-ray images: 68 tons of plastic waste per y; emissions produced by e-waste: 6313-7892 tons of CO_2_ equivalent (135 tons of plastic waste); avoided silver and silver nitrate by digitalizing x-ray images: 4.3 tons of silver per y and 6.8 tons of silver nitrate per y; avoided hydroquinone by digitalizing x-ray images: 26.5 tons per y; avoided water use by digitalizing x-ray images: 270,656,943 L per y; impact on emissions produced by EHRs: positive net effect—1,840,000-2,300,000 tons of CO_2_ equivalent and negative net effect—720,000-900,000 tons of CO_2_ equivalent
Andrew et al [82], Australia, 2020	University hospital (nephrology)	Evaluating the environmental impact of NF2F versus F2F consultations	Telemedicine (NS)	Travel-related emissions	US EPA and GNAF^al^	None	NA	Carbon emissions (CO_2_)	Avoided emissions in total for 263 NF2F consultations: 51,000 kg of CO_2_ equivalent (on 203,202 km traveled)
Dullet et al [83], United States, 2017	University hospital	Evaluating the environmental impact of NF2F versus hypothetical F2F consultations	Telemedicine (NS)	Travel-related emissions	US EPA and MP Mileage 2.5	None	NA	GHG emissions	Avoided emissions in total for 38,051 NF2F consultations (on 8,602,912.51 km traveled): 1,969,000 kg of CO_2_, 50 tons of carbon monoxide, 3.7 tons of nitrogen oxides, and 5.5 tons of volatile organic compounds
Carr and Kevitt [84], Ireland, 2023	Hospital (occupational health)	Evaluating the environmental impact of NF2F versus F2F consultations	Telemedicine (T)	Travel-related emissions	Google Maps and SEAI^am^	None	NA	Carbon emissions (CO_2_)	Avoided emissions in total for 66 NF2F consultations: 305.6 kg of CO_2_ equivalent (on 2029.2 km traveled)
Bove et al [85], United States, 2023	University hospital (neurology)	Evaluating the cost-effectiveness and data quality of NF2F versus F2F disability assessments for patients with multiple sclerosis	Telemedicine (VC)	No	NA	OEC (DT)	Utility Bidder (1080p video at 0.015 kg per h)	Carbon emissions (CO_2_)	Emissions per F2F visit: mean 89 kg of CO_2_; emissions per NF2F visit: mean 0.018 kg of CO_2_
Lathan et al [86], United Kingdom, 2024	Specialized health center (surgery)	Evaluating the environmental impact of NF2F versus F2F postoperative follow-up consultations	Telemedicine (VC)	Building emissions, staff service, medical equipment, and pharmaceuticals	Building emissions: UK DEFRA and UK NIHR; staff service: UK PSSRU^an^; medical equipment: NA; pharmaceuticals: NA	None	NA	Carbon emissions (CO_2_)	Avoided emissions per NF2F consultation: 41.2 kg of CO_2_ equivalent (on a median of 42.5 km traveled); avoided emissions in total for 31 NF2F consultations: 1594 kg of CO_2_ equivalent
Wolf et al [10], United States, 2022	NS (ophthalmology)	To compare the marginal GHG emissions of conducting a diabetic eye examination using autonomous AI^ao^ versus a traditional eye care provider	AI	Diabetic eye examination by an ophthalmologist	US EPA	OEC	US EPA	Carbon emissions (CO_2_)	Emissions per AI diagnostic examination: 0.02-0.2 g of CO_2_ equivalent; total emissions including additional in-person examination: 0.16 kg of CO_2_ equivalent
Mojdehbakhsh et al [87], United States, 2021	Hospital (gynecology)	Evaluating the environmental impact of NF2F versus F2F outpatient gynecologic oncology consultations	Telemedicine (NS)	Travel-related emissions	US EPA	None	NA	Carbon emissions (CO_2_)	Avoided emissions in total for 193 NF2F consultations per week: 6250 kg of CO_2_ equivalent (on 24,964 km traveled)
Sillcox et al [51], United States, 2023	Hospital (gastroenterology)	Evaluating the environmental impact of NF2F versus F2F consultations	Telemedicine (VC)	Travel-related emissions, medical equipment, and paper use	Travel-related emissions: Holmner et al [19] and ecoinvent; medical equipment: LCA and ecoinvent; paper use: NA	OEC (DT and SR)	ecoinvent	Carbon emissions (CO_2_)	Emissions per F2F consultation: 38.22-39.61 kg of CO_2_ equivalent; emissions per NF2F consultation: 2.22-2.9 kg of CO_2_ equivalent
Sillcox et al [88], United States, 2023	Hospital (surgery)	Evaluating the environmental impact of NF2F versus F2F consultations	Telemedicine (VC)	Travel-related emissions	US EPA	None	NA	Carbon emissions (CO_2_)	Emissions per F2F consultation: 201 kg of CO_2_ equivalent; emissions per NF2F consultation: 36.6 kg of CO_2_ equivalent; emissions in total for 51 F2F consultations per y: 10,225 kg of CO_2_ equivalent (on 40,733 km traveled); avoided emissions in total for 55 NF2F consultations per y: 2011.4 kg of CO_2_ equivalent (on 8013 km traveled)
Thota et al [89], United States, 2020	Hospital (oncology)	Evaluating the environmental impact of NF2F versus F2F consultations	Telemedicine (NS)	Travel-related emissions	Google Maps and US EPA	None	NA	Carbon emissions (CO_2_)	Avoided emissions per NF2F patient: 1334 kg of CO_2_ equivalent (in 4 y on 4596 km traveled); avoided emissions in total for 1025 NF2F consultations: 158,673 kg of CO_2_ equivalent (in 4 y on 547,125 km traveled)
Wootton et al [90], Scotland, 2010	Regional health care sector	Evaluating the environmental impact of NF2F visits on reducing travel-related carbon emissions within the NHS	Telemedicine (NS)	Travel-related emissions	UK DEFRA	None	NA	Carbon emissions (CO2)	Avoided emissions in total for 2061 NF2F visits: 59,000 kg of CO_2_ per y (on 260,000 km traveled)
Blenkinsop et al [91], United Kingdom, 2021	University hospital (neurology)	Evaluating the environmental impact of NF2F versus F2F consultations in patients with epilepsy	Telemedicine (VC)	Travel-related emissions	ArcGIS, Google Maps, and UK BEIS	OEC (D and DT)	UK BEIS and Aslan et al [92]	Carbon emissions (CO_2_)	Avoided emissions in total for 1277 NF2F consultations: 35,000-41,088 kg of CO_2_ equivalent; emissions in total for 1277 NF2F consultations: 2 (telephone)-167 (VC) kg of CO_2_ equivalent
Miah et al [93], United Kingdom, 2019	Hospital (urology)	Evaluating the environmental impact of NF2F versus F2F consultations	Telemedicine (NS)	Travel-related emissions	UK DfT^ap^, Google Maps, and Carbon Footprint	None	NA	Carbon emissions (CO_2_)	Avoided emissions in total for 409 NF2F consultations: 350-1450 kg of CO_2_ equivalent (median 3.8 miles traveled); projected avoided emissions in total for 409 NF2F consultations per y: 1050-4350 kg of CO_2_ equivalent
Paquette and Lin [94], United States, 2019	Hospital (surgery)	Evaluating the environmental and financial impacts of NF2F versus F2F consultations	Telemedicine (VC)	Travel-related emissions	Google Maps and US EPA	None	NA	GHG emissions	Avoided emissions in total for 146 NF2F consultations: 1632 kg of CO_2_, 0.043 kg of carbon monoxide, 3.160 kg of nitric oxides, and 4.715 kg of volatile organic compounds
Peters et al [95], United Kingdom, 2020	University hospital (radiology)	Evaluating the environmental impact of travel by radiology trainees and assessing the potential of remote work and tele-radiology	Remote work	Travel-related emissions	ClimateCare	None	NA	Carbon emissions (CO_2_)	Emissions produced by working in person per trainee: 1850 kg of CO_2_ per y (average of 6600 miles per y); avoided emissions with remote (PROC^aq^) setup in total: 4300 kg of CO_2_ equivalent
Bartlett and Keir [96], United Kingdom, 2022	Hospital	Evaluating the environmental impact of NF2F versus F2F consultations during the COVID-19 pandemic	Telemedicine (T and VC)	Travel-related emissions, building emissions, and medical equipment	Travel-related emissions: Google Maps and UK DEFRA; building emissions: UK DEFRA; medical equipment: Rizan et al [97]	Travel-related emissions: OEC; building emissions and medical equipment: EEC	Travel-related emissions: NA; building emissions and medical equipment: LCA (Ong et al [46])	Carbon emissions (CO_2_)	Emissions per F2F consultation: 4.824 kg of CO_2_ equivalent; emissions per NF2F consultation: 0.994 kg of CO_2_ equivalent
Croghan et al [98], Ireland, 2021	Hospital (urology)	Evaluating the environmental impact of NF2F versus F2F consultations in a urological outpatient setting	Telemedicine (VC)	Travel-related emissions	AA Route Planner, Google Maps, and Carbon Footprint	None	NA	Carbon emissions (CO_2_)	Avoided emissions in total for 736 NF2F consultations: 6070 kg of CO_2_ equivalent (49,951 km traveled in 3 mo)
Oliveira et al [99], Portugal, 2013	Specialized health center	Evaluating the environmental impact of NF2F versus F2F appointments	Telemedicine (VC)	Travel-related emissions	Google Maps, UK DEFRA, and UK DECC^ar^	None	NA	Carbon emissions (CO_2_)	Avoided emissions per NF2F consultation: 22 kg of CO_2_ equivalent; avoided emissions in total for 20,824 NF2F consultations: 455,000 kg of CO_2_ equivalent (2,313,819 km traveled in 8 y)
Gupta et al [100], United Kingdom, 2023	Hospital (otolaryngology)	Assessing the effectiveness of and satisfaction with remote telephone consultations for patients referred for potential tonsillectomy	Telemedicine (T)	Travel-related emissions	Google Maps and UK BEIS	None	NA	Carbon emissions (CO_2_)	Avoided emissions for 33 NF2F visits: mean 5.17 kg of CO_2_ equivalent; avoided emissions for 16 NF2F visits: 8.77 kg of CO_2_ equivalent (on a mean of 39.48 km traveled by car); avoided emissions for 11 NF2F visits: 1.31 kg of CO_2_ equivalent (on a mean of 8.86 km traveled by bus); avoided emissions for 3 NF2F visits: 5.35 kg of CO_2_ equivalent (on a mean of 121.2 km traveled by train)
Schulz et al [101], Australia, 2020	Hospital (infectiology)	Evaluating the effectiveness and environmental impact of NF2F versus F2F hepatitis C outreach services	Telemedicine (NS)	Travel-related emissions	BITRE^as^	None	NA	Carbon emissions (CO_2_)	Avoided emissions in total for 58 NF2F consultations: 22,370 kg of CO_2_ equivalent per y
Udayaraj et al [102], United Kingdom, 2019	Hospital (nephrology)	Implementing and evaluating an NF2F clinic service for patients undergoing kidney transplant; environmental impact versus F2F was evaluated	Telemedicine (NS)	Travel-related emissions	National Energy Foundation	None	NA	Carbon emissions (CO_2_)	Avoided emissions in total for 97 NF2F consultations: 1035 kg of CO_2_ equivalent (on 5676.16 km traveled)
Roy et al [103], United States, 2023	University hospital (gastroenterology)	Evaluating the environmental impact of NF2F versus F2F visits	Telemedicine (T and VC)	Travel-related emissions	Google Maps	None	NA	Carbon emissions (CO_2_)	Avoided emissions per NF2F consultation: 31.5 kg of CO_2_ equivalent (average of 126.61 km traveled); avoided emissions in total for 111 NF2F consultations: 3175.15 kg of CO_2_ equivalent (14,038.31 km traveled)

^a^Data: Assumptions and data used for nondigital calculations.

^b^Factors: Methodological assumptions and factors per technology.

^c^LCA: life cycle analysis.

^d^Outcome: Measured environmental income

^e^NF2F: non–face-to-face.

^f^F2F: face-to-face.

^g^T: telephone.

^h^DEFRA: Department for Environment, Food, and Rural Affairs.

^i^NHS: National Health Service.

^j^NA: not assessed.

^k^CO_2_: carbon dioxide.

^l^OSA: obstructive sleep apnea.

^m^OM: otitis media.

^n^VC: videoconference.

^o^GHG: greenhouse gas.

^p^EEC: embodied energy consumption.

^q^OEC: operational energy consumption.

^r^D: device.

^s^DT: data transmission.

^t^SR: system resources.

^u^NS: not specified.

^v^NIHR: National Institute for Health and Care Research.

^w^ISO: International Organization for Standardization.

^x^EPA: Environmental Protection Agency.

^y^IEA: International Energy Agency.

^z^LCI: life cycle inventory.

^aa^EHR: electronic health record.

^ab^TREMOD: Transport Emission Model.

^ac^UBA: German Federal Environment Agency.

^ad^UN: United Nations.

^ae^ICAO: International Civil Aviation Organization.

^af^SPML: selective percutaneous myofascial lengthening.

^ag^BEIS: Department for Business, Energy, and Industrial Strategy.

^ah^FHWA: Federal Highway Administration.

^ai^PM10: particulate matter≤10μm.

^aj^PM2.5: particulate matter≤2.5μm.

^ak^eWCC: Virtual Wound Care Command Centre.

^al^GNAF: Geocoded National Address File.

^am^SEAI: Sustainable Energy Authority of Ireland.

^an^PSSRU: Personal Social Services Research Unit.

^ao^AI: artificial intelligence.

^ap^DfT: UK Department for Transport.

^aq^PROC: Peninsula Radiology On-Call.

^ar^DECC: Department of Energy and Climate Change.

^as^BITRE: Bureau of Infrastructure and Transport Research Economics.

**Figure 2:**
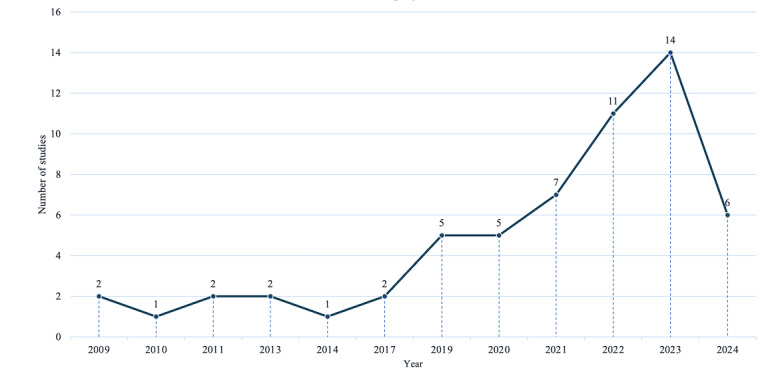
Number of studies by year of publication.

The publication date of the studies included ranged between 2009 and 2024, with a notable increase in the number of publications per year, particularly from 2020 to 2023 (Figure 2). When considering the entire period, this trend is evident as nearly three-quarters (43/58, 74%) of the studies were published during these years [10,43,44,49-53,55-59,61-63,65,68,69,71-77, 79,80,84-88,91,96,98,100,103].

Most of the studies (34/58, 59%) were conducted either in the United States (21/24, 88%; Table 1) or in the United Kingdom (13/24, 54%; Table 1). Other studies were conducted in Australia (3/58, 5%) [77,82,101], Canada (3/58, 5%) [44,52,53], Scotland (3/58, 5%) [54,64,90], Spain (3/58, 5%) [56,70,80], Germany (2/58, 3%) [62,69], Sweden (2/58, 3%) [19,49], and Ireland (2/58, 3%) [84,98]. Studies were also conducted in Wales (1/58, 2%) [60], Portugal (1/58, 2%) [99], Oman (1/58, 2%), Italy (1/58, 2%) [50], Denmark (1/58, 2%) [74], and Switzerland (1/58, 2%) [75].

**Figure 3:**
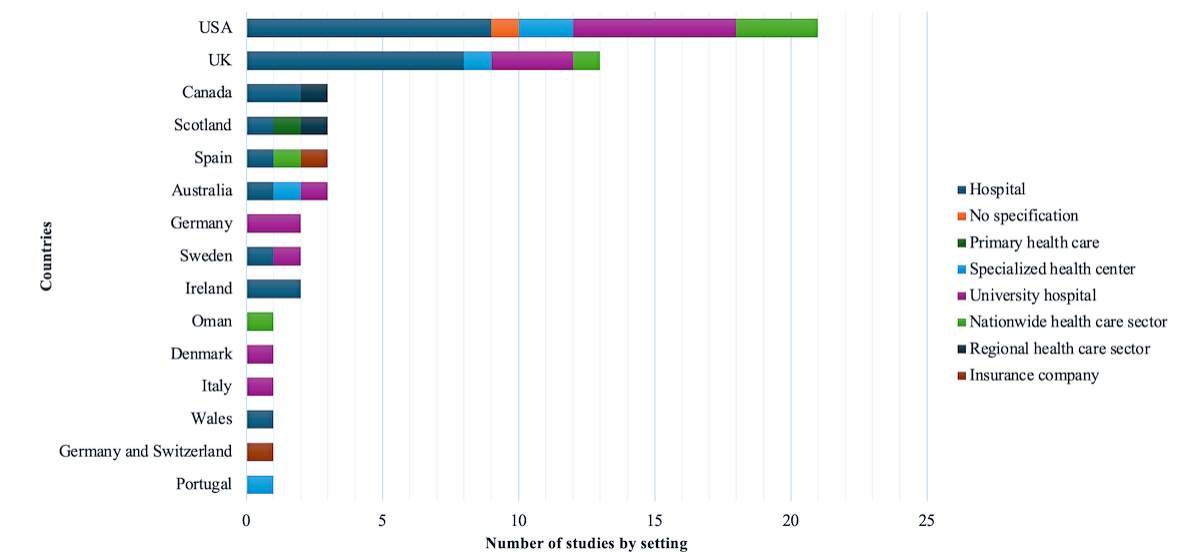
Number of studies by country and study setting. UK: United Kingdom; USA: United States.

Examining the settings of the studies, it is evident that more than two-thirds (41/58, 71%) were conducted in hospital environments, including general hospitals (26/58, 45%; Table 1) and university hospitals (15/58, 26%; Table 1). A smaller number of studies focused on the broader health care system either at the regional (2/58, 3%) [53,90] or national (6/58, 10%) level [47,57,58,65,70,81], encompassing a range of health care settings. Only 2% (1/58) of the studies were conducted in the context of primary health care [54]. Figure 3 shows the number of studies by country and setting for further information.

### Environmental Assessment of Digital Health Technologies

The digital technology most frequently analyzed (Table 2) in terms of environmental sustainability was telemedicine (53/58, 91%). Of these studies, most (33/58, 57%; Table 1) focused on the videoconferencing format, whereas only 10% (6/58) of the studies examined telephony [43,48,57,84,96,100,103] and 5% (3/58) of the studies included both formats. A total of 19% (11/58) of the studies investigating telemedicine did not specify the form of telemedicine analyzed (Table 1). Other digital technologies examined, each in a single study, included AI [10], a digital application [77], a web platform [79], and remote working in radiology [95].

An analysis of the methods used to assess the environmental impact of digital technologies reveals that, regardless of the specific technology examined, nearly all studies (56/58, 97%; Table 1) quantified the transport-related emissions that were avoided through digitalization. A subset of studies (5/58, 9%) [43,47,55,86,96] additionally calculated the emissions avoided from reduced building operations in health care facilities considering factors such as heating, ventilation, air conditioning, lighting, and waste. Furthermore, 5% (3/58) of the studies [51,55,86] included emissions generated by medical equipment such as personal protective equipment, hand sanitizer, and durable medical examination devices. Lathan et al [86] additionally included emissions produced by pharmaceuticals. Wolf et al [10] investigated the greenhouse gas emissions caused by an ophthalmologic examination conducted by an ophthalmologist compared to an examination conducted using AI.

Only 28% (16/58) of the studies [19,44,47,50,55,81,96] compared the energy consumption and associated emissions of the examined digital technologies. All these studies (16/16, 100%) included the energy consumption generated by the use of digital technologies, meaning the operational energy. A wide range of factors were considered in these calculations. The most frequently included factor was end-user devices (8/16, 50%) [19,44,50,56,58,75,81,91], followed by data transmission (6/16, 38%) [19,51,56,58,85,91] and system resource use (5/16, 31%) [19,50,51,58,62], such as network or server use.

Energy consumption data were sourced from government institutions in 8 studies, including the US Environmental Protection Agency (n=3, 38%) [10,55,58]; the German Federal Environment Agency (n=2, 25%) [62,75]; the UK Department for Environment, Food, and Rural Affairs and Department for Business, Energy, and Industrial Strategy (n=2, 25%) [47,91]; and the Government of British Columbia (n=1, 12%) [44]. Only Schmitz-Grosz et al [75] included emissions from building premises alongside energy consumption in their analysis.

**Table 2:** Number of studies by digital technology assessed.

Digital technology assessed	Studies (n=58), n (%)
Remote working	1 (2)
Digital application	1 (2)
Web platform	1 (2)
Artificial intelligence	1 (2)
Electronic health record	2 (3)
Telemedicine	53 (91)

Nearly half (7/16, 44%) of the studies that compared the energy consumption and associated emissions of the examined digital technologies (Table 1) used LCA to quantify emissions generated throughout the life cycle of digital technologies from production to disposal. This represents 12% (7/58) of the total number of studies. The LCA methodology proposed by Ong et al [46] was the most frequently used (3/7, 43%) [19,44,96]. Other frameworks used included the International Organization for Standardization 14040/14044 standard (1/7, 14%) [55], the ecoinvent version 3.8 database (1/7, 14%) [50], and the EcoHealth Footprint framework (1/7, 14%) [81], each applied in a different individual study.

### Reported Outcomes: Environmental, Social, and Economic Impacts

Most studies (47/58, 81%) expressed the environmental impact of digital technologies in terms of avoided CO_2_ emissions. Additional studies (10/58, 17%; Table 1) reported on various greenhouse gas emissions, such as carbon monoxide, nitrogen oxides, and methane. Only Turley et al [81] provided insights beyond avoiding greenhouse gas emissions and discussed the reductions in waste, toxic chemicals, water use, and air pollution resulting from digitalization. However, these calculations were conducted solely for the traditional form of x-ray imaging, not for the digital variant [81].

The CO_2_ emissions avoided using telemedicine ranged from 0.57 kg [75] to 499.84 kg [62] per consultation. For studies that did not report specific data on the avoided emissions per consultation, these were calculated based on the total emissions and the number of consultations. The arithmetic mean of these values was calculated as medians with SDs were not possible to calculate due to the lack of individual values. The calculations were conducted using Microsoft Excel (version 16.95.1). A positive correlation was identified between the amount of emissions avoided and the distance saved by avoiding travel to health care facilities. In total, 830,118,137 kg of CO_2_ were avoided across all telemedicine studies. However, it was not possible to determine the total emissions avoided in 12% (7/58) of the studies [44,47,50-52,85,96]. Barakat-Johnson et al [77] reported a CO_2_ reduction of 250 kg for 51 users of the Virtual Wound Care Command Centre app. Similarly, Peters et al [95] demonstrated that remote work can reduce CO_2_ emissions by up to 4300 kg. The use of the MyPreOp web platform by 813 patients, as analyzed by Milne-Ives et al [79], resulted in CO_2_ savings of 9050 kg over a 4-month period. Wolf et al [10] estimated CO_2_ emissions of 0.02 to 0.2 g for eye examinations conducted using AI technology in contrast to the approximately 8 kg emitted during a traditional ophthalmological examination.

In nearly two-thirds of the studies reviewed (36/58, 62%), social outcomes were also considered in addition to ecological effects (Table 3). Key social end points included patient satisfaction (16/58, 28%; Table 3) and overall patient experience (6/58, 10%) [43,49,61,74,82,102], with additional attention paid to patient safety (2/58, 3%) [54,91] and health outcomes (9/58, 16%; Table 3). In total, 3% (2/58) of the studies [62,64] evaluated the general feasibility of telemedicine across various fields. Another frequently examined end point was the time savings associated with the use of digital technologies (9/58, 16%; Table 3). Only 3% (2/58) of the studies [74,100] incorporated the perspective of health care staff. Additional factors examined included quality-adjusted life years (1/58, 2%) [47], the associations between telehealth use and demographic variables (1/58, 2%) [63], and the impact of telehealth use on low-income families (1/58, 2%) [67].

Economic factors were explored in 48% (28/58) of the studies (Table 3). These investigations encompassed the reduction in travel costs for patients (24/58, 41%; Table 3), loss of income due to attending in-person appointments (6/58, 10%) [52,54,67,69,89,100], and financial savings for health care providers (10/58, 17%; Table 3).

**Table 3:** Studies that assessed social and economic impacts.

Study, country, year	Economic impact	Social impact
Curtis et al [43], United Kingdom, 2021	Yes—travel costs (patient)	Yes—patient preference and patient experience
Heffernan et al [44], Canada, 2023	No	Yes—patient safety
Holmner et al [19], Sweden, 2014	No	No
Smith et al [47], United Kingdom, 2013	Yes—financial costs (health care system)	Yes—QALYs^a^
Connor et al [48], United Kingdom, 2011	Yes—financial costs (health care system)	Yes—patient safety
Kassa et al [49], Sweden, 2024	No	Yes—patient satisfaction and patient experience
Savoldelli et al [50], Italy, 2024	No	No
Tselapedi-Sekeitto et al [52], Canada, 2024	Yes—travel costs (patient) and lost wages	Yes—patient satisfaction
Welk et al [53], Canada, 2022	Yes—travel costs (patient)	No
Dorrian et al [54], Scotland, 2009	Yes—travel costs (patient) and lost wages	Yes—patient satisfaction and patient safety
Thiel et al [55], United States, 2023	No	No
Morcillo Serra et al [56], Spain, 2022	No	No
Jiang et al [57], United States, 2021	Yes—travel costs (patient)	Yes—patient satisfaction
Cummins et al [58], United States, 2024	No	No
Grabski et al [59], United States, 2024	No	No
Lewis et al [60], Wales, 2009	Yes—travel costs (patient)	Yes—time savings
Evers et al [61], United States, 2021	Yes—travel costs (patient)	Yes—patient experience and patient preference
Arndt et al [62], Germany, 2023	No	Yes—patient satisfaction, time savings, and feasibility
Cockrell et al [63], United States, 2022	No	Yes—association between demographics and telehealth use
Sellars et al [64], Scotland, 2020	Yes—travel costs (patient) and financial costs (health care system)	Yes—feasibility
Al Fannah et al [65], Oman, 2022	No	No
Robinson et al [67], United States, 2017	Yes—travel costs (patient) and lost wages	Yes—time savings, caregiver burden, and impact on low-income families
Chang et al [68], United States, 2023	Yes—travel costs (patient)	No
Muschol et al [69], Germany, 2022	Yes—travel costs (patient) and lost wages	No
Vidal-Alaball [70], Spain, 2019	Yes—travel costs (patient)	Yes—time savings
King et al [71], United Kingdom, 2022	No	Yes—health outcomes
Lee et al [72], United States, 2021	No	No
Patel et al [73], United States, 2023	No	No
Dieperink et al [74], Denmark, 2023	No	Yes—time savings, patient experience, and nurse experience
Schmitz-Grosz et al [75], Germany and Switzerland, 2023	No	No
Iaccarino et al [76], United States, 2022	Yes—travel costs (patient)	Yes—patient satisfaction
Barakat-Johnson et al [77], Australia, 2022	Yes—travel costs (patient)	Yes—patient satisfaction and health outcomes
Connor et al [78], United Kingdom, 2019	Yes—financial costs (health care system)	Yes—health outcomes
Milne-Ives et al [79], United Kingdom, 2022	No	Yes—patient satisfaction and time savings
Moncho-Santonja et al [80], Spain, 2023	Yes—travel costs (patient)	No
Turley et al [81], United States, 2011	No	No
Andrew et al [82], Australia, 2020	No	Yes—patient experience
Dullet et al [83], United States, 2017	Yes—travel costs (patient)	Yes—patient satisfaction
Carr and Kevitt [84], Ireland, 2023	No	Yes—patient satisfaction
Bove et al [85], United States, 2023	Yes—travel costs (patient) and financial costs (health care system)	Yes—health outcomes
Lathan et al [86], United Kingdom, 2024	No	No
Wolf et al [10], United States, 2022	No	No
Mojdehbakhsh et al [87], United States, 2021	No	Yes—patient satisfaction
Sillcox et al [51], United States, 2023	No	No
Sillcox et al [88], United States, 2023	No	Yes—health outcomes
Thota et al [89], United States, 2020	Yes—travel costs (patient) and lost wages	Yes—time savings
Wootton et al [90], Scotland, 2010	No	No
Blenkinsop et al [91], United Kingdom, 2021	Yes—travel costs (patient) and financial costs (health care system)	Yes—patient safety
Miah et al [93], United Kingdom, 2019	Yes—financial costs (health care system)	Yes—patient satisfaction
Paquette and Lin [94], United States, 2019	Yes—travel costs (patient)	Yes—time savings
Peters et al [95], United Kingdom, 2020	No	No
Bartlett and Keir [96], United Kingdom, 2022	No	No
Croghan et al [98], Ireland, 2021	Yes—travel costs (patient)	Yes—health outcomes and time savings
Oliveira et al [99], Portugal, 2013	No	No
Gupta et al [100], United Kingdom, 2023	Yes—travel costs (patient) and lost wages	Yes—health outcomes, patient satisfaction, and nurse experience
Schulz et al [101], Australia, 2020	Yes—travel costs (patient) and financial costs (health care system)	Yes—patient satisfaction and health outcomes
Udayaraj et al [102], United Kingdom, 2019	Yes—financial costs (health care system)	Yes—health outcomes, patient satisfaction, and patient experience
Roy et al [103], United States, 2023	No	Yes—patient satisfaction

^a^QALY: quality-adjusted life year.

## Discussion

### The Digitalization of Health Care: More Than Just Telemedicine?

An analysis of the results related to the RQs reveals that the ecological benefits of digitalization in health care are predominantly studied in the context of telemedicine. Rodler et al [18] recently demonstrated, across 48 systematically analyzed studies, that telemedicine is associated with reduced CO_2_ emissions and contributes to lowering the health care sector’s carbon footprint. Similarly, in their review, Purohit et al [104] identified a wide variance in CO_2_ emissions, ranging from 0.70 to 372 kg per consultation. They found that the reduction in CO_2_ emissions resulting from the avoidance of in-person visits was primarily linked to a decrease in travel distance to health care facilities. To explain the considerable variance in these study results, a comparison between the studies by Purohit et al [104] and Rodler et al [18] also reveals that this variance was partly attributable to the underlying modeling approaches used for calculating CO_2_ emissions, as well as the technologies used and the degree of specialization of the facilities [18,104]. According to Rodler et al [18], factors that may play a role in the modeling include not only the consideration of emissions from the technologies used but also the underlying data used for the calculations as these are often based on assumptions and estimates rather than precise measurements. Furthermore, it is clear that data collection and evaluations of AI, electronic health records, and other digital applications in health care have only been conducted to a limited extent.

If we examine the publications in the field of AI, it becomes evident that it can contribute in many different ways to overcoming problems in the health care sector. In addition to assisting with environmental medical challenges such as predicting outbreaks of infectious diseases or real-time analysis of environmental data on air quality [16], AI can also help optimize energy consumption [14]. However, it quickly becomes apparent that research into the environmental impacts associated with the use of AI in health care is scarce. In this review, only 2% (1/58) of the studies examined the carbon footprint of AI [10]. Other studies examining the sustainability of AI generally rely on data concerning its use in broader contexts, such as industry or general applications [105]. Although these studies provide an important initial step in highlighting the potential for AI to promote greater sustainability in health care, they fail to fully address the specific environmental impacts of AI within the health care sector [14,16]. A similar gap exists in the literature regarding the use of digital patient records. Beyond a few studies documenting reductions in energy consumption [106], detailed investigations are lacking, with only 2 comprehensive studies included in this review [56,81]. In addition, only 1 digital health application has been analyzed with respect to its ecological impact [77], further underscoring the significant research deficit in evaluating the environmental consequences of digital health care technologies other than telemedicine.

The question arises of how such a significant research gap regarding the sustainability of other digital health technologies has emerged. When examining the studies by their publication date, it becomes apparent that, during the COVID-19 pandemic (2020-2023), there was an increase in publications in the telemedicine field as there was a heightened demand for web-based services [59,71,74]. Furthermore, telemedicine is one of the longest-established digital technologies, making it likely that it has been studied more frequently. In addition to these aspects, it must also be considered that telemedicine provides very concrete metrics, such as travel distance and mode of transport, which can be used to calculate avoided CO_2_ emissions [74]. Calculating CO_2_ emissions and other environmental impacts of other digital technologies is more challenging due to the complexity of the digital infrastructure. Factors such as the withholding of data by individual companies in the supply chains of digital devices, which complicates comprehensive calculations for hardware and software; the lack of standardization in CO_2_ emission calculation and modeling methods for digital technologies; and the use of different energy sources all play a role in this complexity [81,107].

To comprehensively assess how digitalization in health care impacts the ecological goals of the Planetary Health movement, further studies that extend beyond the evaluation of telemedicine are needed. Data collection for digital technologies such as AI and electronic health records should be conducted specifically within the context of health care. This is essential for a holistic assessment of the environmental effects of these technologies.

### Clean Digital Health?

While the environmental impact of digitalization is most often evaluated through the perspective of CO_2_ emissions, such assessments often overlook other significant factors, including the generation of electronic waste and its associated consequences, as well as the extraction and consumption of natural resources [108,109]. Only 2% (1/58) of the studies in this review, using the EcoHealth Footprint framework, provided a more comprehensive understanding of the environmental impacts of digitalization [81]. Relying solely on CO_2_ emissions as an indicator of environmental sustainability provides an incomplete picture of the true ecological impact of digitalization. Therefore, it is imperative that future research adopts more comprehensive assessment approaches that incorporate a broader range of environmental factors to ensure meaningful and informed evaluations [50,81]. Moyano-Fernández et al [108], along with Thompson [25] and Vandemeulebroucke [110], emphasize the importance of considering the long-term ecological effects of AI and other digital technologies to determine what constitutes sustainable digitalization. Therefore, it is essential to explore how additional environmental effects can be identified and incorporated into ecological assessments [37,38]. One proposed approach for achieving a more holistic and long-term perspective on digitalization is through LCA [38]. However, given that only 12% (7/58) of the studies [19,44,47,50,55,81,96] used LCAs in their assessments, a clear discrepancy emerges between the recognized need for long-term evaluation of digitalization and the LCAs that have actually been conducted. In their review, Lokmic-Tomkins et al [38] examined assessment tools for digital technologies and similarly concluded that no standardized procedure exists for assessing the ecological impact of digital technologies in the health care sector. This highlights the necessity for more consistent and comprehensive approaches to environmental evaluation in this field.

### Planetary Health as a Framework

Previous research has emphasized the need for uniform and stringent framework conditions and guidelines. Bevere and Faccilongo [111] conducted a comprehensive analysis of the application of digital technologies in the health care sector to enhance energy efficiency across various domains. They concluded that there is a pressing need for government initiatives to prioritize energy efficiency and environmental sustainability. In addition to providing tax incentives for environmentally friendly technologies, these initiatives should also incorporate employee training [111].

The establishment of such structures and frameworks at both national and international levels using diverse strategic approaches is deemed essential [12]. Rahimi-Ardabili et al [13] raised concerns regarding the evaluation and assessment of existing tools, particularly in terms of their validity for decision-making at multiple levels concerning sustainability in health care. Furthermore, there is a notable lack of awareness among decision makers regarding established green IT frameworks, leading to the underuse of existing systems [12].

Samuel and Lucassen [109], building on the work by Lannelongue et al [112], developed initial approaches and formulated 10 principles for ecologically sustainable IT practices in the health care sector. These principles not only emphasize the need for documenting CO_2_ emissions associated with IT systems but also advocate for the prolonged use of devices and the judicious selection of equipment with regard to ecological sustainability [109]. Ueda et al [14] proposed action principles for the sustainable use and ecological consideration of AI. Their findings suggest that, in addition to the application of LCA, efforts toward green computing, the development of energy-efficient AI models, and factors such as education and integration into broader sustainability initiatives are equally important [14]. Sittig et al [113] introduced the IT-Enabled Clinical Climate Informatics Actions for the Environment framework, which presents further approaches to creating sustainable conditions, including promoting a circular economy, reducing energy consumption, supporting administrators in decision-making, mobilizing the workforce, and informing employees about policies and regulations to drive change. Sijm-Eeken et al [114] also developed a framework that combined medical informatics solutions with their environmental impacts. The *Green Mission framework* incorporates IT solution architecture (enhanced by governance), climate resilience, and environmental impacts [114].

On the basis of a first cursory comparison of these different frameworks for sustainable digitalization, the following factors should be considered when implementing such a guideline [14,109,113,114]: (1) long-term perspective regarding the sustainability of deployed technologies, (2) interoperability of digital systems, (3) stakeholder engagement and cooperation among various actors in health care, (4) sociotechnical dimensions for integrating digital technologies into daily work routines, (5) sustainability as a multidimensional concept, (6) data protection and security, and (7) improving health care delivery through the use of digital technologies.

Despite the consideration of numerous factors, it remains apparent that there are still several gaps regarding technical feasibility, social inclusion, and evaluative measures.

A comprehensive analysis of these frameworks with regard to their practical applicability, existing gaps, and long-term effectiveness could contribute to the development of a more integrated and holistic concept for sustainable digitalization within the health care sector [38,115].

The findings related to social and economic aspects reflect the core principles of the Planetary Health concept, aiming to integrate sustainability, social benefits, and economic considerations within a holistic framework [115]. For example, digital health solutions that reduce travel-related emissions may also improve patient convenience and access—especially for marginalized populations—while alleviating pressure on health care infrastructure. However, the limited inclusion of health care staff perspectives (only 2/58, 3% of the studies) and sparse attention to economic metrics such as quality-adjusted life years (1/58, 2% of the studies) suggest an underexplored opportunity to further integrate all 3 dimensions of sustainability in future research [116,117].

It is evident that the implementation of ecologically sustainable digitalization is highly complex and requires consideration of a wide range of factors aligned with the goals of sustainable health, as advocated by the Planetary Health movement [109,114]. However, a standardized assessment framework or set of guidelines for achieving sustainable digitalization in the health care sector has yet to be identified [38].

Recently, Ip [118] outlined the social ambivalence of digitalization in relation to the goals of the Planetary Health movement in a commentary published in *The Lancet*. The ethical guidelines derived from this discussion emphasize, first, the need for equitable access to digitalization, the preservation of natural ecosystems, and the significant responsibility placed on professionals to engage in sustainable practices [118].

Building on this, the ecological ambivalence of digitalization, as outlined previously, underscores the necessity for guidelines that promote ecologically sustainable digitalization in the health care sector [25]. Given that the Planetary Health movement not only addresses ecological sustainability but also integrates human health and social issues, thereby offering a broader conception of sustainability, it serves as an ideal foundation for developing a comprehensive set of guidelines.

Thus, Planetary Health can provide the guiding framework for the creation of appropriate directives for sustainable digital transformation in health care.

### Limitations

Potential limitations of this study include the risk of bias due to the involvement of a single reviewer (single-reviewer bias). As the primary selection and evaluation of studies were conducted by a single reviewer, there is a possibility that personal biases, individual experiences, or methodological preferences impacted the objectivity of the results [119]. To mitigate this, eligible and critical studies were discussed in collaboration with a second reviewer. However, to further address this limitation, future research should involve multiple independent reviewers to ensure a more balanced and robust interpretation of the data. Another limitation arises from the absence of a systematic assessment of study quality. In addition, some full-text articles were inaccessible or excluded due to language barriers, potentially leading to the exclusion of relevant publications from this review.

### Conclusions

There have already been efforts to make the increasing digitalization of the health care sector ecologically sustainable, and certain digital technologies have demonstrated ecologically sustainable effects. However, a clear discrepancy exists between the available studies assessing the ecological impact of digitalization in health care and the growing demand for ecological guidelines. This is evident in the lack of comprehensive assessments, such as LCA, or the consideration of factors beyond CO_2_ emissions in evaluating ecological impacts. Furthermore, there is a noticeable scarcity of actionable strategies and their evaluations in terms of ecological sustainability in the existing literature. In conclusion, the following key points can be highlighted:

1. The need for research into the environmental sustainability of digital technologies in health care is underscored by the health care sector’s significant annual contribution to greenhouse gas emissions.
2. Research is required to investigate the ecological impact of digital technologies in health care beyond the scope of telemedicine. While digital technologies such as AI, electronic health records, and digital applications are highly relevant for the health care sector both at present and in the future, it is essential to intensify and deepen research in this area with regard to their sustainability. Environmental assessments that adopt a holistic approach, incorporating LCA; supply chain considerations; and, more broadly, social, ecological, and economic factors, should be prioritized.
3. There is a pressing need for actionable strategies to ensure the sustainable implementation of digitalization in health care with a comprehensive evaluation of its ecological impact.
4. Planetary Health should serve as the guiding framework for developing action-oriented guidelines for ecological sustainability in digitalization to address and mitigate the inherent ambivalence of digital transformation.

## Appendix

Multimedia Appendix 1 PRISMA-ScR (Preferred Reporting Items for Systematic Reviews and Meta-Analyses: Scoping Review) checklist.

Multimedia Appendix 2 Search string.

## Abbreviations

AI: artificial intelligence
CO_2_: carbon dioxide
LCA: life cycle analysis
PRISMA: Preferred Reporting Items for Systematic Reviews and Meta-Analyses
RQ: research question

## References

[1] Nikendei C, Bugaj TJ, Nikendei F, Kühl SJ, Kühl M (2020). Klimawandel: Ursachen, Folgen, Lösungsansätze und Implikationen für das Gesundheitswesen. Z Evid Fortbild Qual Gesundhwes.

[2] Surface air temperature for September 2024. Copernicus Climate Change Service.

[3] State of the global climate 2023; WMO-No. 1347. World Meteorological Organization.

[4] Romanello M, Napoli CD, Green C, Kennard H, Lampard P, Scamman D, Walawender M, Ali Z, Ameli N, Ayeb-Karlsson S, Beggs PJ, Belesova K, Berrang Ford L, Bowen K, Cai W, Callaghan M, Campbell-Lendrum D, Chambers J, Cross TJ, van Daalen KR, Dalin C, Dasandi N, Dasgupta S, Davies M, Dominguez-Salas P, Dubrow R, Ebi KL, Eckelman M, Ekins P, Freyberg C, Gasparyan O, Gordon-Strachan G, Graham H, Gunther SH, Hamilton I, Hang Y, Hänninen R, Hartinger S, He K, Heidecke J, Hess JJ, Hsu S, Jamart L, Jankin S, Jay O, Kelman I, Kiesewetter G, Kinney P, Kniveton D, Kouznetsov R, Larosa F, Lee JK, Lemke B, Liu Y, Liu Z, Lott M, Lotto Batista M, Lowe R, Odhiambo Sewe M, Martinez-Urtaza J, Maslin M, McAllister L, McMichael C, Mi Z, Milner J, Minor K, Minx JC, Mohajeri N, Momen NC, Moradi-Lakeh M, Morrissey K, Munzert S, Murray KA, Neville T, Nilsson M, Obradovich N, O'Hare MB, Oliveira C, Oreszczyn T, Otto M, Owfi F, Pearman O, Pega F, Pershing A, Rabbaniha M, Rickman J, Robinson EJ, Rocklöv J, Salas RN, Semenza JC, Sherman JD, Shumake-Guillemot J, Silbert G, Sofiev M, Springmann M, Stowell JD, Tabatabaei M, Taylor J, Thompson R, Tonne C, Treskova M, Trinanes JA, Wagner F, Warnecke L, Whitcombe H, Winning M, Wyns A, Yglesias-González M, Zhang S, Zhang Y, Zhu Q, Gong P, Montgomery H, Costello A (2023). The 2023 report of the Lancet Countdown on health and climate change: the imperative for a health-centred response in a world facing irreversible harms. Lancet.

[5] Romanello M, McGushin A, Di Napoli C, Drummond P, Hughes N, Jamart L, Kennard H, Lampard P, Solano Rodriguez B, Arnell N, Ayeb-Karlsson S, Belesova K, Cai W, Campbell-Lendrum D, Capstick S, Chambers J, Chu L, Ciampi L, Dalin C, Dasandi N, Dasgupta S, Davies M, Dominguez-Salas P, Dubrow R, Ebi KL, Eckelman M, Ekins P, Escobar LE, Georgeson L, Grace D, Graham H, Gunther SH, Hartinger S, He K, Heaviside C, Hess J, Hsu S, Jankin S, Jimenez MP, Kelman I, Kiesewetter G, Kinney PL, Kjellstrom T, Kniveton D, Lee JK, Lemke B, Liu Y, Liu Z, Lott M, Lowe R, Martinez-Urtaza J, Maslin M, McAllister L, McMichael C, Mi Z, Milner J, Minor K, Mohajeri N, Moradi-Lakeh M, Morrissey K, Munzert S, Murray KA, Neville T, Nilsson M, Obradovich N, Sewe MO, Oreszczyn T, Otto M, Owfi F, Pearman O, Pencheon D, Rabbaniha M, Robinson E, Rocklöv J, Salas RN, Semenza JC, Sherman J, Shi L, Springmann M, Tabatabaei M, Taylor J, Trinanes J, Shumake-Guillemot J, Vu B, Wagner F, Wilkinson P, Winning M, Yglesias M, Zhang S, Gong P, Montgomery H, Costello A, Hamilton I (2021). The 2021 report of the Lancet Countdown on health and climate change: code red for a healthy future. Lancet.

[6] Semenza JC, Suk JE (2018). Vector-borne diseases and climate change: a European perspective. FEMS Microbiol Lett.

[7] Baker RE, Mahmud AS, Miller IF, Rajeev M, Rasambainarivo F, Rice BL, Takahashi S, Tatem AJ, Wagner CE, Wang L, Wesolowski A, Metcalf CJ (2022). Infectious disease in an era of global change. Nat Rev Microbiol.

[8] (2018). COP24 special report: health and climate change. World Health Organization.

[9] Kariner J, Slotterback S, Boyd R, Ashby B, Steele K (2019). Health cares climate footprint: how the health sector contributes to the global climate crisis and opportunities for action. Health Care Without Harm.

[10] Wolf RM, Abramoff MD, Channa R, Tava C, Clarida W, Lehmann HP (2022). Potential reduction in healthcare carbon footprint by autonomous artificial intelligence. NPJ Digit Med.

[11] Tennison I, Roschnik S, Ashby B, Boyd R, Hamilton I, Oreszczyn T, Owen A, Romanello M, Ruyssevelt P, Sherman JD, Smith AZ, Steele K, Watts N, Eckelman MJ (2021). Health care's response to climate change: a carbon footprint assessment of the NHS in England. Lancet Planet Health.

[12] Holmner A, Rocklöv J, Ng N, Nilsson M (2012). Climate change and eHealth: a promising strategy for health sector mitigation and adaptation. Glob Health Action.

[13] Rahimi-Ardabili H, Magrabi F, Coiera E (2022). Digital health for climate change mitigation and response: a scoping review. J Am Med Inform Assoc.

[14] Ueda D, Walston SL, Fujita S, Fushimi Y, Tsuboyama T, Kamagata K, Yamada A, Yanagawa M, Ito R, Fujima N, Kawamura M, Nakaura T, Matsui Y, Tatsugami F, Fujioka T, Nozaki T, Hirata K, Naganawa S (2024). Climate change and artificial intelligence in healthcare: review and recommendations towards a sustainable future. Diagn Interv Imaging.

[15] Klimaeffekte Der Digitalisierung 2.0 - Bitkom - Accenture- 1.0: Studie Zur Abschätzung Des Beitrags Digitaler Technologien Zum Klimaschutz in Deutschland. Bitkom.

[16] Zaidan AM (2023). The leading global health challenges in the artificial intelligence era. Front Public Health.

[17] Lo Man C, Lin Y, Pang G, Sanderson J, Duan K (2024). Digitalization to achieve greener healthcare supply chain. J Clean Prod.

[18] Rodler S, Ramacciotti LS, Maas M, Mokhtar D, Hershenhouse J, de Castro Abreu AL, Fuchs G, Stief CG, Gill IS, Cacciamani GE (2023). The impact of telemedicine in reducing the carbon footprint in health care: a systematic review and cumulative analysis of 68 million clinical consultations. Eur Urol Focus.

[19] Holmner A, Ebi KL, Lazuardi L, Nilsson M (2014). Carbon footprint of telemedicine solutions--unexplored opportunity for reducing carbon emissions in the health sector. PLoS One.

[20] Tidman R, Abela-Ridder B, de Castañeda RR (2021). The impact of climate change on neglected tropical diseases: a systematic review. Trans R Soc Trop Med Hyg.

[21] Liao H, Lyon CJ, Ying B, Hu T (2024). Climate change, its impact on emerging infectious diseases and new technologies to combat the challenge. Emerg Microbes Infect.

[22] Ranjan Laha S, Pattanayak BK, Pattnaik S (2022). Advancement of environmental monitoring system using IoT and sensor: a comprehensive analysis. AIMS Environ Sci.

[23] Neo EX, Hasikin K, Mokhtar MI, Lai KW, Azizan MM, Razak SA, Hizaddin HF (2022). Towards integrated air pollution monitoring and health impact assessment using federated learning: a systematic review. Front Public Health.

[24] Ansari M, Alam M (2023). An intelligent IoT-cloud-based air pollution forecasting model using univariate time-series analysis. Arab J Sci Eng.

[25] Thompson M (2021). The environmentally impacts of digital health. Digit Health.

[26] Belkhir L, Elmeligi A (2018). Assessing ICT global emissions footprint: trends to 2040 and recommendations. J Clean Prod.

[27] Turk D, Cozzi L Digitalization and energy. International Energy Agency.

[28] Andrae A, Edler T (2015). On global electricity usage of communication technology: trends to 2030. Challenges.

[29] Sovacool BK, Ali SH, Bazilian M, Radley B, Nemery B, Okatz J, Mulvaney D (2020). Sustainable minerals and metals for a low-carbon future. Science.

[30] The global e-waste monitor 2024. International Telecommunication Union, Fondation Carmignac, United Nations Institute for Training and Research.

[31] Berg H, Ramesohl S (2019). Nachhaltigkeit und Digitale Transformation: Bericht zum Forschungsmodul B1 Wechselwirkungen des Ziel- und Indikatorensystems. Wuppertal Institut für Klima.

[32] Perkins DN, Brune Drisse MN, Nxele T, Sly PD (2014). E-waste: a global hazard. Ann Glob Health.

[33] Whitmee S, Haines A, Beyrer C, Boltz F, Capon AG, de Souza Dias BF, Ezeh A, Frumkin H, Gong P, Head P, Horton R, Mace GM, Marten R, Myers SS, Nishtar S, Osofsky SA, Pattanayak SK, Pongsiri MJ, Romanelli C, Soucat A, Vega J, Yach D (2015). Safeguarding human health in the Anthropocene epoch: report of the Rockefeller Foundation-Lancet Commission on planetary health. Lancet.

[34] Planetary health: an emerging field to be developed. Royal Netherlands Academy of Arts and Science.

[35] Herrmann A, Lenzer B, Müller BS, Danquah I, Nadeau KC, Muche-Borowski C, Traidl-Hoffmann C (2022). Integrating planetary health into clinical guidelines to sustainably transform health care. Lancet Planet Health.

[36] Pongsiri MJ, Bickersteth S, Colón C, DeFries R, Dhaliwal M, Georgeson L, Haines A, Linou N, Murray V, Naeem S, Small R, Ungvari J (2019). Planetary health: from concept to decisive action. Lancet Planet Health.

[37] Savoldelli A, Landi D, Rizzi C (2023). Sustainability in healthcare: methods and tools for the assessment. Stud Health Technol Inform.

[38] Lokmic-Tomkins Z, Davies S, Block LJ, Cochrane L, Dorin A, von Gerich H, Lozada-Perezmitre E, Reid L, Peltonen LM (2022). Assessing the carbon footprint of digital health interventions: a scoping review. J Am Med Inform Assoc.

[39] Page MJ, McKenzie JE, Bossuyt PM, Boutron I, Hoffmann TC, Mulrow CD, Shamseer L, Tetzlaff JM, Akl EA, Brennan SE, Chou R, Glanville J, Grimshaw JM, Hróbjartsson A, Lalu MM, Li T, Loder EW, Mayo-Wilson E, McDonald S, McGuinness LA, Stewart LA, Thomas J, Tricco AC, Welch VA, Whiting P, Moher D (2021). The PRISMA 2020 statement: an updated guideline for reporting systematic reviews. BMJ.

[40] Tricco AC, Lillie E, Zarin W, O'Brien KK, Colquhoun H, Levac D, Moher D, Peters MD, Horsley T, Weeks L, Hempel S, Akl EA, Chang C, McGowan J, Stewart L, Hartling L, Aldcroft A, Wilson MG, Garritty C, Lewin S, Godfrey CM, Macdonald MT, Langlois EV, Soares-Weiser K, Moriarty J, Clifford T, Tunçalp Ö, Straus SE (2018). PRISMA extension for scoping reviews (PRISMA-ScR): checklist and explanation. Ann Intern Med.

[41] Richardson WS, Wilson MC, Nishikawa J, Hayward RS (1995). The well-built clinical question: a key to evidence-based decisions. ACP J Club.

[42] Faster systematic literature reviews. rayyan.

[43] Curtis A, Parwaiz H, Winkworth C, Sweeting L, Pallant L, Davoudi K, Smith E, Chin K, Kelsey M, Stevenson A (2021). Remote clinics during coronavirus disease 2019: lessons for a sustainable future. Cureus.

[44] Heffernan A, Lalande A, Chadha R, MacNeill A, Chadha NK (2024). Carbon savings potential of virtual care in obstructive sleep apnea and otitis media with effusion. Laryngoscope Investig Otolaryngol.

[45] Lenzen M (1999). Total requirements of energy and greenhouse gases for Australian transport. Transp Res D Transp Environ.

[46] Ong D, Moors T, Sivaraman V (2012). Complete life-cycle assessment of the energy/CO2 costs of videoconferencing vs face-to-face meetings. Proceedings of the 2012 IEEE Online Conference on Green Communications.

[47] Smith AJ, Tennison I, Roberts I, Cairns J, Free C (2013). The carbon footprint of behavioural support services for smoking cessation. Tob Control.

[48] Connor A, Mortimer F, Higgins R (2011). The follow-up of renal transplant recipients by telephone consultation: three years experience from a single UK renal unit. Clin Med (Lond).

[49] Kassa AM, Nyström N, Waldenvik K, Engstrand Lilja H (2024). Experiences and satisfaction of video follow up of children with paediatric gastrointestinal conditions linking tertiary centre with guardians and clinicians at the local hospital: a cross-sectional study. BMC Pediatr.

[50] Savoldelli A, Landi D, Rizzi C (2024). Exploring the environmental impact of telemedicine: a life cycle assessment. Stud Health Technol Inform.

[51] Sillcox R, Gitonga B, Meiklejohn DA, Wright AS, Oelschlager BK, Bryant MK, Tarefder R, Khan Z, Zhu J (2023). The environmental impact of surgical telemedicine: life cycle assessment of virtual vs. in-person preoperative evaluations for benign foregut disease. Surg Endosc.

[52] Tselapedi-Sekeitto B, Rocha T, Sowerby LJ, Rotenberg B, Biadsee A (2023). Telemedicine as an environmental ally - the social, financial, and environmental impact of virtual care in the otolaryngology clinic. Am J Otolaryngol.

[53] Welk B, McArthur E, Zorzi AP (2022). Association of virtual care expansion with environmental sustainability and reduced patient costs during the COVID-19 pandemic in Ontario, Canada. JAMA Netw Open.

[54] Dorrian C, Ferguson J, Ah-See K, Barr C, Lalla K, van der Pol M, McKenzie L, Wootton R (2009). Head and neck cancer assessment by flexible endoscopy and telemedicine. J Telemed Telecare.

[55] Thiel CL, Mehta N, Sejo CS, Qureshi L, Moyer M, Valentino V, Saleh J (2023). Telemedicine and the environment: life cycle environmental emissions from in-person and virtual clinic visits. NPJ Digit Med.

[56] Morcillo Serra C, Aroca Tanarro A, Cummings CM, Jimenez Fuertes A, Tomás Martínez JF (2022). Impact on the reduction of CO2 emissions due to the use of telemedicine. Sci Rep.

[57] Jiang CY, Strohbehn GW, Dedinsky RM, Raupp SM, Pannecouk BM, Yentz SE, Ramnath N (2021). Teleoncology for veterans: high patient satisfaction coupled with positive financial and environmental impacts. JCO Oncol Pract.

[58] Cummins MR, Shishupal S, Wong B, Wan N, Han J, Johnny JD, Mhatre-Owens A, Gouripeddi R, Ivanova J, Ong T, Soni H, Barrera J, Wilczewski H, Welch BM, Bunnell BE (2024). Travel distance between participants in US telemedicine sessions with estimates of emissions savings: observational study. J Med Internet Res.

[59] Grabski DF, Meyer MJ, Gander JW (2024). Pediatric telemedicine visits reduce greenhouse gas emissions. J Clim Chang Health.

[60] Lewis D, Tranter G, Axford AT (2009). Use of videoconferencing in Wales to reduce carbon dioxide emissions, travel costs and time. J Telemed Telecare.

[61] Evers EC, Fritz SA, Colditz GA, Burnham JP (2022). Perceptions of telemedicine and costs incurred by a visit to a general infectious diseases clinic: a survey. Open Forum Infect Dis.

[62] Arndt EM, Jansen TR, Bojko J, Roos JJ, Babasiz M, Randau TM, Welle K, Burger C, Kabir K (2023). COVID-19 measures as an opportunity to reduce the environmental footprint in orthopaedic and trauma surgery. Front Surg.

[63] Cockrell HC, Maine RG, Hansen EE, Mehta K, Salazar DR, Stewart BT, Greenberg SL (2022). Environmental impact of telehealth use for pediatric surgery. J Pediatr Surg.

[64] Sellars H, Ramsay G, Sunny A, Gunner CK, Oliphant R, Watson AJ (2020). Video consultation for new colorectal patients. Colorectal Dis.

[65] Al Fannah J, Al Sabahi S, Al Harthi H, Al Bahrani M, Al Salmi Q (2023). Towards a green hospital approach in Oman: a case study of quantifying an environmental impact. Int J Health Plann Manage.

[66] Ravindrane R, Patel J (2022). The environmental impacts of telemedicine in place of face-to-face patient care: a systematic review. Future Healthc J.

[67] Robinson JD, Prochaska JD, Yngve DA (2017). Pre-surgery evaluations by telephone decrease travel and cost for families of children with cerebral palsy. SAGE Open Med.

[68] Chang JH, Maskal SM, Ellis RC, Prabhu AS, Rosen MJ, Walsh RM, Miller BT (2023). Zooming to net zero: using virtual visits to decrease carbon emissions and costs from surgery. J Gastrointest Surg.

[69] Muschol J, Heinrich M, Heiss C, Hernandez AM, Knapp G, Repp H, Schneider H, Thormann U, Uhlar J, Unzeitig K, Gissel C (2022). Economic and environmental impact of digital health app video consultations in follow-up care for patients in orthopedic and trauma surgery in Germany: randomized controlled trial. J Med Internet Res.

[70] Vidal-Alaball J, Franch-Parella J, Lopez Seguí F, Garcia Cuyàs F, Mendioroz Peña J (2019). Impact of a telemedicine program on the reduction in the emission of atmospheric pollutants and journeys by road. Int J Environ Res Public Health.

[71] King J, Poo SX, El-Sayed A, Kabir M, Hiner G, Olabinan O, Colwill M, Ayubi H, Shakweh E, Kronsten VT, Kader R, Hayee B, GLINT Research Network (2023). Towards NHS Zero: greener gastroenterology and the impact of virtual clinics on carbon emissions and patient outcomes. A multisite, observational, cross-sectional study. Frontline Gastroenterol.

[72] Lee J, Yousaf A, Jenkins S, Zaki MT, Napier C, Abdul-Aziz OI, Zinn Z (2021). The positive environmental impact of virtual isotretinoin management. Pediatr Dermatol.

[73] Patel KB, Gonzalez BD, Turner K, Alishahi Tabriz A, Rollison DE, Robinson E, Naso C, Wang X, Spiess PE (2023). Estimated carbon emissions savings with shifts from in-person visits to telemedicine for patients with cancer. JAMA Netw Open.

[74] Dieperink KB, Vestergaard LV, Møller PK, Tolstrup LK (2023). Using video consultations for clinical assessment and decision of treatment readiness before chemotherapy: a mixed-methods study among patients with gastrointestinal cancer and oncology nurses. Digit Health.

[75] Schmitz-Grosz K, Sommer-Meyer C, Berninger P, Weiszflog E, Jungmichel N, Feierabend D, Battegay E (2023). A telemedicine center reduces the comprehensive carbon footprint in primary care: a monocenter, retrospective study. J Prim Care Community Health.

[76] Iaccarino MA, Paganoni S, Tenforde A, Silver JK, Schneider JC, Slocum C, Polak R, Alexander M, Hefner J (2022). Environmental impact of telerehabilitation visits in an urban setting. J Clim Chang Health.

[77] Barakat-Johnson M, Kita B, Jones A, Burger M, Airey D, Stephenson J, Leong T, Pinkova J, Frank G, Ko N, Kirk A, Frotjold A, White K, Coyer F (2022). The viability and acceptability of a virtual wound care command centre in Australia. Int Wound J.

[78] Connor MJ, Miah S, Edison MA, Brittain J, Smith MK, Hanna M, El-Husseiny T, Dasgupta R (2019). Clinical, fiscal and environmental benefits of a specialist-led virtual ureteric colic clinic: a prospective study. BJU Int.

[79] Milne-Ives M, Leyden J, Maramba I, Chatterjee A, Meinert E (2022). The potential impacts of a digital preoperative assessment service on appointments, travel-related carbon dioxide emissions, and user experience: case study. JMIR Perioper Med.

[80] Moncho-Santonja M, Aparisi-Navarro S, Defez Garcia B, Davol A, Peris-Fajarnés G (2022). Health care in rural areas: proposal of a new telemedicine program assisted from the reference health centers, for a sustainable digitization and its contribution to the carbon footprint reduction. Heliyon.

[81] Turley M, Porter C, Garrido T, Gerwig K, Young S, Radler L, Shaber R (2011). Use of electronic health records can improve the health care industry's environmental footprint. Health Aff (Millwood).

[82] Andrew N, Barraclough KA, Long K, Fazio TN, Holt S, Kanhutu K, Hughes PD (2020). Telehealth model of care for routine follow up of renal transplant recipients in a tertiary centre: a case study. J Telemed Telecare.

[83] Dullet NW, Geraghty EM, Kaufman T, Kissee JL, King J, Dharmar M, Smith AC, Marcin JP (2017). Impact of a university-based outpatient telemedicine program on time savings, travel costs, and environmental pollutants. Value Health.

[84] Carr P, Kevitt F (2023). Service user satisfaction with telemedicine in an occupational healthcare setting. Occup Med (Lond).

[85] Bove R, Poole S, Cuneo R, Gupta S, Sabatino J, Harms M, Cooper T, Rowles W, Miller N, Gomez R, Lincoln R, McPolin K, Powers K, Santaniello A, Renschen A, Bevan CJ, Gelfand JM, Goodin DS, Guo C, Romeo AR, Hauser SL, Campbell Cree BA (2023). Remote observational research for multiple sclerosis: a natural experiment. Neurol Neuroimmunol Neuroinflamm.

[86] Lathan R, Hitchman L, Walshaw J, Ravindhran B, Carradice D, Smith G, Chetter I, Yiasemidou M (2024). Telemedicine for sustainable postoperative follow-up: a prospective pilot study evaluating the hybrid life-cycle assessment approach to carbon footprint analysis. Front Surg.

[87] Mojdehbakhsh RP, Rose S, Peterson M, Rice L, Spencer R (2021). A quality improvement pathway to rapidly increase telemedicine services in a gynecologic oncology clinic during the COVID-19 pandemic with patient satisfaction scores and environmental impact. Gynecol Oncol Rep.

[88] Sillcox R, Blaustein M, Khandelwal S, Bryant MK, Zhu J, Chen JY (2023). Telemedicine use decreases the carbon footprint of the bariatric surgery preoperative evaluation. Obes Surg.

[89] Thota R, Gill DM, Brant JL, Yeatman TJ, Haslem DS (2020). Telehealth is a sustainable population health strategy to lower costs and increase quality of health care in rural Utah. JCO Oncol Pract.

[90] Wootton R, Tait A, Croft A (2010). Environmental aspects of health care in the Grampian NHS region and the place of telehealth. J Telemed Telecare.

[91] Blenkinsop S, Foley A, Schneider N, Willis J, Fowler HJ, Sisodiya SM (2021). Carbon emission savings and short-term health care impacts from telemedicine: an evaluation in epilepsy. Epilepsia.

[92] Aslan J, Mayers K, Koomey Jg, France C (2017). Electricity intensity of internet data transmission: untangling the estimates. J Ind Ecol.

[93] Miah S, Dunford C, Edison M, Eldred-Evans D, Gan C, Shah T, Lunn P, Winkler M, Ahmed H, Gibbons N, Hrouda D (2019). A prospective clinical, cost and environmental analysis of a clinician-led virtual urology clinic. Ann R Coll Surg Engl.

[94] Paquette S, Lin JC (2019). Outpatient telemedicine program in vascular surgery reduces patient travel time, cost, and environmental pollutant emissions. Ann Vasc Surg.

[95] Peters S, Burrows S, Jenkins P (2021). The challenge of environmental sustainability in radiology training and potential solutions. Postgrad Med J.

[96] Bartlett S, Keir S (2022). Calculating the carbon footprint of a geriatric medicine clinic before and after COVID-19. Age Ageing.

[97] Rizan C, Reed M, Bhutta MF (2021). Environmental impact of personal protective equipment distributed for use by health and social care services in England in the first six months of the COVID-19 pandemic. J R Soc Med.

[98] Croghan SM, Rohan P, Considine S, Salloum A, Smyth L, Ahmad I, Lynch T, Manecksha R (2021). Time, cost and carbon-efficiency: a silver lining of COVID era virtual urology clinics?. Ann R Coll Surg Engl.

[99] Oliveira TC, Barlow J, Gonçalves L, Bayer S (2013). Teleconsultations reduce greenhouse gas emissions. J Health Serv Res Policy.

[100] Gupta T, Bowles P, Bhutta MF (2024). Effectiveness, perceptions and environmental benefits of remote consultation for adults referred with recurrent tonsillitis. Ann R Coll Surg Engl.

[101] Schulz TR, Kanhutu K, Sasadeusz J, Watkinson S, Biggs BA (2020). Using telehealth to improve access to hepatitis C treatment in the direct-acting antiviral therapy era. J Telemed Telecare.

[102] Udayaraj UP, Watson O, Ben-Shlomo Y, Langdon M, Anderson K, Power A, Dudley C, Evans D, Burhouse A (2019). Establishing a tele-clinic service for kidney transplant recipients through a patient-codesigned quality improvement project. BMJ Open Qual.

[103] Roy W, Hans B, Jannat RU, Reddy YK, Hadi Y, Gayam S (2023). Tele-visits for GERD: "ecofriendly, efficient and effective". J Gastroenterol Hepatol.

[104] Purohit A, Smith J, Hibble A (2021). Does telemedicine reduce the carbon footprint of healthcare? A systematic review. Future Healthc J.

[105] Bloomfield P, Clutton-Brock P, Pencheon E, Magnusson J, Karpathakis K (2021). Artificial intelligence in the NHS: climate and emissions. J Clim Chang Health.

[106] García-Berná JA, Fernández-Alemán JL, Carrillo de Gea JM, Toval A, Mancebo J, Calero C, García F (2021). Energy efficiency in software: a case study on sustainability in personal health records. J Clean Prod.

[107] Sadeghi R. K, Qaisari Hasan Abadi M (2024). Sustainable supply chain resilience for logistics problems: empirical validation using robust and computational intelligence methods. J Clean Prod.

[108] Moyano-Fernández C, Rueda J, Delgado J, Ausín T (2024). May Artificial Intelligence take health and sustainability on a honeymoon? Towards green technologies for multidimensional health and environmental justice. Glob Bioeth.

[109] Samuel G, Lucassen AM (2023). The environmental impact of data-driven precision medicine initiatives. Camb Prism Precis Med.

[110] Vandemeulebroucke T (2025). The ethics of artificial intelligence systems in healthcare and medicine: from a local to a global perspective, and back. Pflugers Arch.

[111] Bevere D, Faccilongo N (2024). Shaping the future of healthcare: integrating ecology and digital innovation. Sustainability.

[112] Lannelongue L, Grealey J, Bateman A, Inouye M (2021). Ten simple rules to make your computing more environmentally sustainable. PLoS Comput Biol.

[113] Sittig DF, Sherman JD, Eckelman MJ, Draper A, Singh H (2022). i-CLIMATE: a "clinical climate informatics" action framework to reduce environmental pollution from healthcare. J Am Med Inform Assoc.

[114] Sijm-Eeken ME, Arkenaar W, Jaspers M, Peute L (2022). Medical informatics and climate change: a framework for modeling green healthcare solutions. J Am Med Inform Assoc.

[115] Alami H, Rivard L, Lehoux P, Ag Ahmed MA, Fortin J, Fleet R (2023). Integrating environmental considerations in digital health technology assessment and procurement: stakeholders' perspectives. Digit Health.

[116] Mago A, Dhali A, Kumar H, Maity R, Kumar B (2024). Planetary health and its relevance in the modern era: a topical review. SAGE Open Med.

[117] Pham LT, Kumar P, Dahana WD, Nguyen HD (2024). Advancing sustainable development through planetary health – a holistic approach to global health: A systematic review. Environ Sci Policy.

[118] Ip EC (2024). Digital planetary health needs ethical guidance. Lancet Planet Health.

[119] Higgins JP, Thomas J (2024). Cochrane Handbook for Systematic Reviews of Interventions. 5th edition.

[120] DeepL Translate. DeepL.

[121] ChatGPT. OpenAI.

