# Supplementary material for: Aligning With the Goals of the Planetary Health Concept Regarding Ecological Sustainability and Digital Health: Scoping Review
Source: J Med Internet Res. 2025 May 28;27:e71795. doi: 10.2196/71795 (PMC12159557; doi:10.2196/71795)
Supplement: Multimedia Appendix 2 [file jmir_v27i1e71795_app2.docx]

# Multimedia Appendix 2: Search string

| **Database** | **Searchstring** | **Date** | **Results** |
| --- | --- | --- | --- |
| **PubMed** | **(planetary health[Title/Abstract] OR climate change[Title/Abstract] OR footprint*[Title/Abstract] OR emission*[Title/Abstract] OR environmental impact*[Title/Abstract] OR environmental sustainability[Title/Abstract] OR global warming[Title/Abstract]) AND (digital transformation[Title/Abstract] OR digital health[Title/Abstract] OR artificial intelligence[Title/Abstract] OR telemedicine[Title/Abstract] OR digital technology[Title/Abstract] OR machine learning[Title/Abstract] OR digitalisation[Title/Abstract] OR e-health[Title/Abstract]) AND (healthcare[Title/Abstract] OR health care[Title/Abstract] OR health*care system[Title/Abstract] OR care[Title/Abstract] OR medicine[Title/Abstract) NOT Positron emission tomography** | **14.08.2024** | **261 Results** |
| **MEDLINE** | **(((planetary health or climate change or footprint* or emission* or environmental impact* or environmental sustainability or global warming) and (digital transformation or digital health or artificial intelligence or telemedicine or digital technology or machine learning or digitalisation or e-health) and (healthcare or health care or health*care*system or care or medicine)) not positron emission tomography).ti,ab.** | **14.08.2024** | **234 Results** |
| **Scopus** | **TITLE-ABS("planetary health" OR "climate change" OR footprint* OR emission* OR "environmental impact*" OR "environmental sustainability" OR"climate protection" OR "global warming" )AND TITLE-ABS("digital transformation" OR "digital health" OR "artificial intelligence" OR telemedicine OR "digital technology" OR "machine learning" OR "e-health") AND TITLE-ABS("healthcare" OR "health care" OR health*care system OR care OR medicine) AND NOT TITLE-ABS("Positron emission tomography")** | **14.08.2024** | **277 Results** |
